# Genome Assembly and Annotation of Soft-Shelled Adlay (*Coix lacryma-jobi* Variety *ma-yuen*), a Cereal and Medicinal Crop in the Poaceae Family

**DOI:** 10.3389/fpls.2020.00630

**Published:** 2020-05-18

**Authors:** Sang-Ho Kang, Byeollee Kim, Beom-Soon Choi, Hyun Oh Lee, Nam-Hoon Kim, Seung Jae Lee, Hye Sik Kim, Myung Ju Shin, Hyo-Won Kim, Kyunghyun Nam, Kyoung Dae Kang, Soo-Jin Kwon, Tae-Jin Oh, Sang-Choon Lee, Chang-Kug Kim

**Affiliations:** ^1^Genomics Division, National Institute of Agricultural Sciences, Rural Development Administration, Jeonju, South Korea; ^2^Department of Life Science and Biochemical Engineering, Graduate School, Sun Moon University, Asan, South Korea; ^3^Phyzen Co., Seongnam, South Korea; ^4^DNA Link, Inc., Seoul, South Korea

**Keywords:** adlay, *Coix lacryma-jobi* variety *ma-yuen*, soft-shelled adlay, genome assembly, seed-specific genes, coixin, benzoxazinoid biosynthesis

## Abstract

*Coix lacryma-jobi*, also called adlay or Job’s tears, is an annual herbal plant belonging to the Poaceae family that has been cultivated as a cereal and medicinal crop in Asia. Despite its importance, however, genomic resources for better understanding this plant species at the molecular level and informing improved breeding strategies remain limited. To address this, we generated a draft genome of the *C. lacryma-jobi* variety *ma-yuen* (soft-shelled adlay) Korean cultivar, Johyun, by *de novo* assembly, using PacBio and Illumina sequencing data. A total of 3,362 scaffold sequences, 1.28 Gb in length, were assembled, representing 82.1% of the estimated genome size (1.56 Gb). Genome completeness was confirmed by the presence of 91.4% of the BUSCO angiosperm genes and mapping ratio of 98.3% of Illumina paired-end reads. We found that approximately 77.0% of the genome is occupied by repeat sequences, most of which are *Gypsy* and *Copia*-type retrotransposons, and evidence-based genome annotation predicts 39,574 protein-coding genes, 85.5% of which were functionally annotated. We further predict that soft-shelled adlay diverged from a common ancestor with sorghum 9.0–11.2 MYA. Transcriptome profiling revealed 3,988 genes that are differentially expressed in seeds relative to other tissues, of which 1,470 genes were strongly up-regulated in seeds and the most enriched Gene Ontology terms were assigned to carbohydrate and protein metabolism. In addition, we identified 76 storage protein genes including 18 seed-specific coixin genes and 13 candidate genes involved in biosynthesis of benzoxazinoids (BXs) including coixol, a unique BX compound found in *C. lacryma-jobi* species. The characterization of those genes can further our understanding of unique traits of soft-shelled adlay, such as high seed protein content and medicinal compound biosynthesis. Taken together, our genome sequence data will provide a valuable resource for molecular breeding and pharmacological study of this plant species.

## Introduction

*Coix lacryma-jobi* is an annual herbal plant belonging to the Poaceae family that is commonly called adlay or Job’s tears. Four varieties of this species are known, including *C. lacryma-jobi* variety (var.) *lacryma-jobi*, *ma-yuen*, *puellarum*, and *stenocarpa*. Among these, *C. lacryma-jobi* var. *lacryma-jobi* is a common wild variety with hard shells that is used as beads for necklaces and rosaries (hereafter, hard-shelled adlay). In contrast, *C. lacryma-jobi* var. *ma-yuen* (hereafter, soft-shelled adlay) is a cultivated variety with soft shells. This plant, which is also called soft-shelled adlay, yi yi (China), and yulmu (Korea), is widely grown as a cereal and medicinal crop in Asian countries, including China, Japan, and Korea (Arora, 1977; Chen et al., 2006; Rural Development administration [RDA], 2013; Zhu, 2017).

The *Coix* genus consists of 9 to 11 species with different levels of ploidy; cultivated *C. lacryma-jobi* is tetraploid (2*n* = 20), with a basic chromosome number of *x* = 5 (Clayton, 1981; Christopher and Benny, 1990; Rao and Nirmala, 2011). *C. lacryma-jobi* is closely related to maize (*Zea mays* ssp. *mays*), sorghum (*Sorghum bicolor*), and foxtail millet (*Setaria italica*), all of which belong to the Panicoideae subfamily in the Poaceae family (Kellogg and Watson, 1993; Kang et al., 2018a). The origin of *Coix* species has been traced to Southeast Asia (Arora, 1977), whereas maize and sorghum originate from America and Africa, respectively (Piperno et al., 2009; Winchell et al., 2018).

*C. lacryma-jobi* seeds contain ∼67% starch and ∼20% protein. In particular, the seed protein content of this species is the highest among cereal crops. The major component (∼79%) of *C. lacryma-jobi* seed proteins is coixin, a type of seed storage prolamin (Ottoboni et al., 1990; Lee et al., 2011; Corke et al., 2016; Zhu, 2017). In addition, seed extracts from this plant have been reported to show various pharmacological activities, such as anti-cancer, antioxidant, anti-inflammatory, anti-allergic, anti-diabetes, and gastroprotective effects (Zhu, 2017). *C. lacryma-jobi* is therefore considered to be a valuable cereal crop, with the ability to supply protein nutrition and medicinal components that are deficient in other cereal species.

*C. lacryma-jobi* can produce several secondary metabolites, including benzoxazinoids (BXs), lactams, and phytosterols (Nagao et al., 1985; Choi et al., 2019). BXs are known to play a prominent role in plant defense against various biotic stresses and in interactions with the surrounding environment (Makowska et al., 2015; Zhou et al., 2018; Choi et al., 2019; Kudjordjie et al., 2019). One BX compound in particular, known as coixol, is of interest due to the fact that it is unique in *C. lacryma-jobi* (Zhu, 2017) and displays various physiological activities, including pharmacological effects (Chen et al., 2011; Lee et al., 2015; Choi et al., 2017). The BX biosynthesis pathway and related genes have been well studied in Poaceae species, such as maize and wheat (Makowska et al., 2015; Zhou et al., 2018). However, this pathway and its associated genes have not been characterized in *C. lacryma-jobi* due to the lack of genomic resources for this species.

Most studies of *C. lacryma-jobi* have focused on its pharmacological effects as a medicinal crop, whereas genetic, molecular and genomic studies of this species have been less focused. Until now, cytogenetic analyses (Han et al., 2010; Cai et al., 2014), genetic linkage map development (Qin et al., 2005), bacterial artificial chromosome (BAC) library construction (Meng et al., 2010), characterization of coixin genes (Ottoboni et al., 1993; Meng et al., 2010; Zhou et al., 2010), and transcriptome analysis (Kang et al., 2018b) have been reported. Most recently, the genome of hard-shelled adlay was characterized (Liu et al., 2019). Although these data have revealed numerous insights into *C. lacryma-jobi* biology, and this species continues to attract interest as an alternative cereal crop with high protein content (Ottoboni et al., 1990), current genomic resources for *C. lacryma-jobi* varieties, in particular, var. *ma-yuen* (soft-shelled adlay), are insufficient for breeding and molecular-based investigations, as compared to other Panicoideae species, such as maize, sorghum, and foxtail millet, whose genomes have been sequenced (Bennetzen et al., 2012; Zhang et al., 2012; Jiao et al., 2017; McCormick et al., 2018). To address this, the adlay genome sequencing project was initiated in 2018 as a part of the National Agricultural Genome Program (NAGP^1^) in Korea. The aim of this initiative is to determine the genome sequence, genetic map, and transcriptome profile of soft-shelled adlay and thereby support comprehensive studies for molecular breeding, biotechnological approaches, and pharmacological research on this plant species.

Here, we present a draft genome sequence and a description of the genomic features of soft-shelled adlay. In addition, we analyze the evolutionary history, as well as the genes contributing to seed protein content and BX biosynthesis in this species. We anticipate that the genomic data generated from this study will provide a valuable resource for molecular breeding and pharmacological study to enhance our understanding of this valuable species and related cereal crops.

## Materials and Methods

### Plant Materials and Extraction of Genomic DNA and RNA

For genomic DNA preparation, young leaves were collected from plants of soft-shelled adlay cultivar Johyun that were bred and maintained by Gyeonggido Agricultural Research & Extension Services^2^ (cultivar registration no. SJ9203) in Korea (Jang et al., 2005). Genomic DNA was extracted from young leaves with the modified cetyltrimethylammonium bromide (CTAB) method (Allen et al., 2006). The quantity and quality of genomic DNA were determined by agarose gel electrophoresis and analysis with an Agilent 2100 Bioanalyzer (Agilent Technologies, United States).

For preparation of total RNA, various tissue samples were collected from the soft-shelled adlay cultivar Johyun plants grown by Gyeonggido Agricultural Research & Extension Services, including the youngest leaves, stems, roots, and flowers (pistils and stamens), as well as early seeds of 98-day-old plants and late seeds of 159-day-old plants. The samples were frozen immediately in liquid nitrogen and stored at −80°C until use. Total RNAs were extracted using the RNeasy Plant Mini kit (QIAGEN, Germany), according to the manufacturer’s instructions. The quality and quantity of RNA samples were determined using Agilent 2100 Bioanalyzer (Agilent Technologies).

### Genome and Transcriptome Sequencing

For Illumina next-generation sequencing (NGS) of genomic DNA, three paired-end (PE) genomic DNA libraries, with insert sizes of 350 bp, 550 bp, and 750 bp, were constructed according to standard Illumina PE library protocols and sequenced using the Illumina HiSeq 2500 platform (Illumina, United States) at Macrogen Co. (Seoul, South Korea). In addition, five mate-pair (MP) genomic DNA libraries, with insert sizes of 3 kb, 5 kb, 8 kb, 10 kb, and 15 kb, were constructed and sequenced using the Illumina HiSeq 2000 platform at Macrogen Co. and Theragen Etex Bio Institute (Suwon, South Korea) (Supplementary Table S1). Low-quality reads and adapter sequences were removed from raw PE and MP sequencing data, using Trimmomatic ver. 0.38 (Bolger et al., 2014), with parameters modified to remove all reads with an average base quality < 20 and those shorter than 50 bases. In addition, duplicate reads from MP sequencing data were removed, using clc_remove_duplicates ver. 4.3.0.^3^

For PacBio sequencing of genomic DNA, a SMRTbell library was constructed with the SMRTbell Template Prep Kit 1.0 (PN 100-259-100), according to the manufacturer’s instructions (Pacific Biosciences, United States). Small SMRTbell template fragments were removed, using the BluePippin Size Selection system with size selection criteria of >12 kb for large-insert libraries (Sage Science, United States), and the constructed library was validated by analysis with an Agilent 2100 Bioanalyzer (Agilent Technologies). After a sequencing primer is annealed to the SMRTbell template, DNA polymerase is bound to the SMRTbell-adaptor complex, using the DNA/Polymerase Binding Kit P6 (Pacific Biosciences). This polymerase-SMRTbell-adaptor complex is then loaded into SMRT cells. The SMRTbell library was sequenced in 71 SMRT cells by C4 chemistry (DNA sequencing Reagent 4.0; Pacific Biosciences); 1 × 240-min movies were captured for each SMRT cell, using the PacBio RS II platform (Pacific Biosciences) at DNA Link Co. (Seoul, South Korea) and Macrogen Co.

For transcriptome analysis, RNA-Sequencing (RNA-Seq) libraries with an insert size of approximately 300 bp were constructed using the Illumina TruSeq RNA sample preparation kit, according to the manufacturer’s instructions. A total of 15 RNA-Seq libraries were prepared for three independent biological replicates of leaves, roots, early seeds, and late seeds and for single samples of stems and flowers. Afterward, pooled RNA-Seq libraries were sequenced by the Illumina HiSeq 2500 platform, with PE reads of 101 bp, at DNA Link Co. Low-quality reads and adaptor sequences were removed using Trimmomatic ver. 0.38, with the modified parameters described above, to obtain high-quality reads (Supplementary Table S2).

For PacBio single-molecule long-read isoform sequencing (Iso-Seq) analysis, the mixture of total RNAs from cultivar Johyun tissue samples, including leaves, roots, and seeds, was sequenced in eight SMRT cells by P6-C4 chemistry, using the PacBio RSII platform at DNA Link Co. Sequences were identified by the SMRT Analysis v2.3 RS_IsoSeq.1 classify protocol, and all full-length reads derived from the same isoform were clustered and polished into consensus sequences, using the TOFU pipeline (isoseq-tofu) (Gordon et al., 2015). PCR chimeras of consensus sequences were also removed with an in-house script (Supplementary Table S2).

### Genome Assembly, Scaffolding, and Validation

The genome size of soft-shelled adlay cultivar Johyun was estimated by *k*-mer frequency analyses. In brief, *k*-mer frequency data of trimmed PE data was obtained using JELLYFISH ver. 2.0 (Marçais and Kingsford, 2011) with an optimal *k*-mer value of 17 and genome size was estimated using a formula (genome size = *k*-mer coverage/mean *k*-mer depth). In addition, the *k*-mer frequency data were further analyzed by findGSE ver. 1.0 (Sun H. et al., 2018), and GenomeScope (Vurture et al., 2017) for genome size estimation. From all estimated genome sizes, the median value of 1.56 Gb was selected and used in this study (Supplementary Figure S1). Based on this genome size, PacBio genome sequencing reads with approximately 95× coverage were generated and *de novo* assembled into contig sequences, using FALCON-Unzip ver. 0.7.0 (Chin et al., 2016) at DNA Link Co. First, primary sequences and associated contig sequences were assembled by FALCON with modified parameters for length cutoff specified based on a subread N50 value of 11 kb (length_cutoff, 11000; length_cutoff_pr, 10000; pa_HPCdaligner_option, -t16 -M32 -e.70 -s100 -k19 -h480 -w8 -l2400 -H11000; ovlp_HPCdaligner_option, -t32 -M32 -e.96 -s100 -k17 -h120 -w5 -l1200 -H10000; and the rest as defaults). Primary and haplotig (a contig of unzippable or phaseable regions of the genome) contig sequences were then phased by Unzip module on FALCON-Unzip ver. 0.7.0 for phased diploid assembly and polished with the consensus algorithm of Quiver ver. 2.3.3 (Chin et al., 2013). Primary and haplotig contig sequences were error-corrected by the Burrows-Wheeler Aligner (BWA) ver. 0.7.12 (Li and Durbin, 2009) with a BWA-MEM algorithm and the rest parameters as defaults, as well as the HaplotypeCaller and FastaAlternateReferenceMaker modules in the Genome Analysis Toolkit (GATK) ver. 3.5^4^ with default parameters, using PE data (∼88×), in order to improve the quality of genome assembly results, as described in previous reports (Yang et al., 2017; Sun G. et al., 2018; Goh et al., 2019). Finally, two contig sets, the primary contig set (8,546 contigs) and the haplotig contig set (13,691 contigs), were generated (Supplementary Table S3).

The primary contig set was selected to generate scaffold sequences (the first version of the scaffold set) using MP reads by SSPACE ver. 3.0 (Boetzer et al., 2011) with default parameters. Among these first-version scaffold sequences, mis-scaffolding sequences were found by re-mapping MP reads onto the scaffold sequences, known as the Assembly Error Correction (AEC) method, and then split into initial contig sequences, in order to generate the second version of the scaffold set. Second-version scaffold sequences were then merged by SSPACE-LongRead ver. 1.1 (Boetzer and Pirovano, 2014), using PacBio long reads with default parameters. These were again corrected by the AEC method, in order to generate the third version of the scaffold set. Third-version scaffold sequences were gap-filled with PE reads, using GapFiller ver. 1.11 (Boetzer and Pirovano, 2012), with default parameters, and then corrected again by re-mapping PE reads and variant calling with GATK ver. 4.0.3.0 as described above. The final scaffold set of 3,362 sequences was generated and designated as a draft genome ver. 1.0 of soft-shelled adlay (Supplementary Table S4).

Genome assembly completeness was validated by analysis with Benchmarking Universal Single-Copy Orthologs (BUSCO) ver. 3.0.2 (Simão et al., 2015), using the lineage dataset, embryophyta_odb9. In addition, genome assembly was also validated by mapping PE data and calculating mapping ratio and genome coverage, using BWA ver. 0.7.17, with default parameters.

### Genome Annotation

Repeat sequences in the draft genome were analyzed using RepeatModeler ver. 1.0.8^5^ and RepeatMasker ver. 4.0.5.^6^ Briefly, consensus repeat sequences in the draft genome were identified and characterized with RepeatModeler, and then were combined with known repeat sequences deposited in RepBase ver.28.04.^7^ Based on the combined sequences, repeat sequences were identified in the genome using RepeatMasker.

Gene-coding regions were predicted in the draft genome sequence with the annotation pipeline illustrated in Supplementary Figure S2. Evidence for the identification of gene-coding regions was first collected from *de novo* and genome-guided transcript sequences assembled from the transcriptome data generated in this study (Supplementary Table S2). Additional evidence was obtained from analysis of amino acid sequence similarity with gene products of maize (database ver. 92.7^8^), sorghum (ver. 3.1.1^9^), rice (*Oryza sativa* ssp. *japonica*, IRGSP-1.0^10^), and *Arabidopsis thaliana* (TAIR10^11^). In addition, *C. lacryma-jobi* protein sequences deposited in GenBank^12^ were also used as evidence.

The first gene prediction was performed based on the evidence described above, using MAKER2 ver. 2.31.8 (Holt and Yandell, 2011), and then the first training dataset was prepared using SNAP ver. 2006-07-28 (Zaharia et al., 2011). Subsequently, the second gene prediction was performed with MAKER2, and the second training dataset was prepared using AUGUSTUS ver. 3.3.2 (Stanke et al., 2006). Finally, the third gene prediction was performed with MAKER2, and gene sequences with an annotation evidence distance (AED) score of 1, which indicates there is no evidence to support the annotation, were removed to improve annotation quality. Among the 226,210 genes with an AED < 1, sequences showing high similarity with transposon-related sequences (transposon, transposase, *gag*, *Copia*, and *Gypsy*) were removed based on conserved domain searches against the Pfam database ver. 32.0, using InterProScan ver. 5.34-73.0 (Jones et al., 2014) and similarity searches of the National Center for Biotechnology Information (NCBI) non-redundant (nr) protein database with BLASTP analysis (*E*-value cutoff of 1e-5). As a result, 47,263 genes were selected and designated as the soft-shelled adlay gene set ver. 1.0. Conserved domain analysis of this gene set was performed again with InterProScan ver. 5.34-73.0, using all databases except the PANTHER database, and then an additional 7,698 transposon-related sequences were further removed to generate gene set ver. 1.1, consisting of 39,565 protein-coding genes. We then manually confirmed and curated the structures of some genes based on evidence for gene-coding regions to generate the final gene set, ver. 1.2, which consists of 39,574 protein-coding genes, and this was used for all further analyses.

### Functional Annotation of Protein-Coding Genes

Sequences with similarity to the soft-shelled adlay gene products were identified using BLASTP analyses (*E*-value cutoff 1e-5) by searching against the NCBI nr protein database. Based on these BLASTP results, Gene Ontology (GO) terms were assigned to soft-shelled adlay gene products, using Blast2GO Command Line ver. 1.4.1 (Götz et al., 2008) and GO database ver. 2018.07. Kyoto Encyclopedia of Genes and Genomes (KEGG) pathway analysis was also performed for soft-shelled adlay gene products with the KEGG Automatic Annotation Server (KAAS^13^), using the single-directional best hit (SBH) method and representative gene sets from both eukaryotes and monocots. In addition, protein sequences homologous to the soft-shelled adlay gene products were identified in sorghum, maize, rice, and *A. thaliana*, using BLASTP analysis (*E*-value cutoff of 1e-5). Conserved domains within predicted protein sequences were further detected using InterProScan ver. 5.34-73.0, with all databases except the PANTHER database.

Genes encoding putative transcription factors (TFs), transcriptional regulators (TRs), and protein kinases (PKs) were identified and classified based on conserved domain structure, using the iTAK ver. 1.7a stand-alone program (Zheng et al., 2016). These proteins were then compared with those found in other plant species.

### Expression Profiling of Protein-Coding Genes

Gene expression profiles were determined by mapping trimmed high-quality RNA-Seq reads onto the soft-shelled adlay genome sequence, using HISAT2 ver. 2.1.0 (Kim et al., 2015) with modified parameters (spliced alignment options, dta-cufflinks; the rest as defaults), and counting the RNA reads mapped to coding sequences (CDSs) with HTSeq-count ver. 0.10.0 (Anders et al., 2015) with modified parameters (stranded, no; the rest as defaults). Fragments per kilobase of transcript per million mapped reads (FPKM) values were calculated based on the number of mapped RNA reads, and these were used to compare gene expression profiles.

### Comparative and Evolutionary Analysis of the Soft-Shelled Adlay Cultivar Johyun Genome With Other Plant Species

A total of 24 plant species with fully sequenced genomes, including 16 Poaceae species, were selected for comparative analysis – nine in the subfamilies Panicoideae, Arundinoideae, Chloridoideae, Micrairoideae, Aristidoideae, and Danthonioideae (PACMAD) clade, seven in the subfamily Bamboos, Oryzoideae, and Pooideae (BOP) clade, and eight other dicotyledon species (Supplementary Table S5). Genes (protein-coding sequences) and predicted protein sequences in these plant species were also analyzed as described above for functional annotation, and the functional annotation information was compared with that of soft-shelled adlay genes. Gene sequences unique to soft-shelled adlay and those shared with sorghum, maize, foxtail millet, and rice were identified by gene clustering, using the OrthoVenn2 web tool (Xu et al., 2019), with plant group parameters and an *E*-value cutoff of 1e-5.

We then estimated the divergence time of soft-shelled adlay within the Poaceae family. For this analysis, single-copy orthologous genes within the gene sets from soft-shelled adlay, sorghum, maize, foxtail millet, and rice were identified by gene clustering analysis, using the OrthoVenn2 web tool. From these, we selected 630 genes in soft-shelled adlay and their orthologs in sorghum, maize, foxtail millet, and rice with similarly sized CDSs and performed divergence time calculations. Briefly, multiple sequence alignments of concatenated sequences were generated using MAFFT ver. 7 (Katoh and Standley, 2013) with default parameters. Divergence time was then estimated by BEAST2 ver. 2.4.3 (Bouckaert et al., 2014), with General Time Reversible (GTR) substitution and a strict clock model (default parameter). In addition, GTR substitution and a relaxed clock model were also applied to estimate divergence time. Tree priors were calibrated using a Yule prior model, and divergence time was calibrated using reported divergence times for the split between foxtail millet-maize and rice (95% confidence interval [CI]: 42–52 million years ago [MYA]) and between maize and sorghum (CI: 9.39–12.47 MYA) from the TimeTree database (Kumar et al., 2017). The resulting tree was visualized by FigTree ver. 1.4.3.^14^

### Identification of Genes Differentially Expressed in Seeds

Genes differentially expressed (DE) in seeds were identified by comparison of RNA-Seq expression data from seeds (i.e., early seeds and late seeds) to expression levels in other non-seed tissues (i.e., leaf, root, stem, and flower), using the Bioconductor package DESeq ver. 1.22.1 (Anders and Huber, 2010) with modified parameters (estimateDispersions method, blind; fitType, local; sharingMode, fit-only; the rest as defaults). Genes showing expression changes ≥ 2-fold, with an adjusted *P*-value < 0.05, were selected, and GO enrichment analysis for these DE genes was performed using the Fisher’s exact test with the following parameters: fold-change (selected genes to total genes) ≥ 2.0, *P*-value < 0.01, and false discovery rate (FDR) < 0.01, provided by Blast2GO ver. 5.2.

### Identification of Genes Encoding Storage Proteins

The Molecular Function GO term, nutrient reservoir activity (GO:0045735), is assigned to genes encoding storage proteins, such as glutelin, napin/bra allergen, high molecular weight (HMW) glutenin, gliadin/low molecular weight (LMW) glutenin, zein seed storage protein, cupin, vegetative storage protein, and albumin. Therefore, soft-shelled adlay genes assigned to this GO term were selected as putative storage protein-encoding genes from GO analysis results obtained from InterProScan ver. 5.34-73.0. A heatmap was generated from the FPKM values of predicted storage protein-encoding genes, using the R-package, pheatmap ver. 1.0.12^15^ with modified parameters (scaling among samples, clustering distance of Euclidean, clustering method of complete and the rest as defaults). In addition, soft-shelled adlay gene products with a conserved zein seed storage protein domain (InterPro Entry ID IPR002530, Pfam PF01559^16^), as identified by InterProScan ver. 5.34-73, were classified as coixin genes. These were compared to other plant gene products with zein seed storage protein domains by performing multiple sequence alignments of the predicted amino acid sequences in MUSCLE with default parameters (included in MEGA ver. 7.0) and generating phylogenetic trees with the maximum-likelihood (ML) method and 1,000 bootstraps, using MEGA ver. 7.0 (Kumar et al., 2016) with default parameters.

### Identification of Genes Involved in BX Biosynthesis

Candidate genes encoding enzymes involved in BX biosynthesis, including indole-3-glycerol phosphate lyase (benzoxazinless 1; BX1), cytochrome P450 monooxygenases (BX2, BX3, BX4, and BX5), 2,4-dihydroxy-1,4-benzoxazin-3-one-glucoside dioxygenase (BX6), 2,4,7-trihydroxy-1,4-benzoxazin-3-one-glucoside 7-*O*-methyltransferase (BX7), UDP-glucosyltransferases (BX8 and BX9), O-methyltransferase (BX10), and β-glucosidase (Glu1/Glu2), were investigated by KAAS analysis, as described above, and genome-wide BLASTP searches (*E*-value cutoff of 1e-4) with maize genes for BX biosynthesis as queries (Makowska et al., 2015; Cotton et al., 2019). In addition, candidate TF genes regulating BX biosynthesis genes were also investigated by genome-wide BLASTP searches (*E*-value cutoff of 1e-4) with two maize basic helix-loop-helix (bHLH) TFs, ZmbHLH20 and ZmbHLH76, known as positive regulators of BX gene expression (Gao et al., 2019). Finally, found genes were confirmed again by construction of a phylogenetic tree after multiple alignment of those protein sequences with corresponding gene family members of soft-shelled adlay, sorghum, maize, foxtail millet, rice, and *A. thaliana*.

### Quantification of 6-Methoxy-1,3-Benzoxazol-2-One (MBOA, Coixol) Using HPLC Analysis

Powders (20 g) of leaves, stems, roots, early seeds and late seeds that were the same as those used for transcriptome analysis were prepared using an electric blender and freeze-drier and extracted with 200 ml of 70% methanol for 2 days using an electromagnetic stirrer. After filtration, extracts were concentrated on a rotatory evaporator under vacuum pump and chromatographed on an Agilent 1100 series HPLC system with Shimadzu shim-pack GIS-ODS column (4.6 mm × 150 mm, 5 um). The mobile phase consisted of acetonitrile (A) and 5% aqueous formic acid solution (B). The flow rate was maintained at 1 ml/min and samples were eluted with the following gradient: 5–15% B (0–10 min), 15–25% B (15–40 min), 25–50% B (15–40 min), 50–80% B (40–50 min) and 80–5% B (50–58 min). Chromatograms were recorded at 280 nm. A coixol standard was purchased from ChemFaces Biochemical Co. Ltd. (Wuhan, China) and used to generate a calibration curve for quantification of coixol. HPLC analyses were performed with at least two independent biological samples.

## Results and Discussion

### Genome Assembly and Annotation of Soft-Shelled Adlay (*C. lacryma-jobi* var. *ma-yuen* ‘Johyun’)

PacBio reads of 148.3 Gb (∼95× coverage) and Illumina PE reads of 136.5 Gb (∼88× coverage) were assembled into 8,546 primary contigs by *de novo* assembly with error-correction (Supplementary Table S3). Using Illumina MP and PacBio long reads, these contig sequences were merged into 3,362 scaffold sequences (Table 1 and Supplementary Table S4), totaling 1.28 Gb in length, with an N50 of 594.3 kb; this was designated as the soft-shelled adlay draft genome ver. 1.0. Sequences in this draft cover 82.1% of the estimated genome size (1.56 Gb, Supplementary Figure S1) of soft-shelled adlay cultivar Johyun. Completeness of the draft genome sequence was confirmed by BUSCO analysis, which found that 1,316 (91.4%) out of 1,440 conserved orthologous angiosperm genes were completely assembled (Supplementary Table S6). In addition, the genome sequence was further validated by mapping PE data, demonstrating that 98.3% of PE reads are covered by the genome sequences (Supplementary Table S7). Thus, these analyses indicate that the genome sequence assembled in this study is of good quality.

**TABLE 1:** Genome assembly and annotation of soft-shelled adlay cultivar Johyun.

**Genome assembly**	
Number of scaffolds	3,362
Total length of scaffolds (bp)	1,280,016,620
N50 of scaffolds (bp)	594,251
Average scaffold length (bp)	380,731
Longest scaffold (bp)	2,711,986
Complete BUSCO (%)	91.4
**Genome annotation**	
Repeat content (%)	77.0
Gene prediction	
Total predicted gene number	39,574
Total length of coding sequences (bp)	47,806,527
Average gene length (bp)	1,208
Minimum gene length (bp)	102
Maximum gene length (bp)	15,927
GC content (%)	55.3
Complete BUSCO (%)	84.6
Functionally annotated (%)	85.5

Approximately 986 Mb (77.0%) of the genome was identified as repeat sequences, of which long terminal repeat (LTR) retrotransposons, including *Gypsy* and *Copia*, are the most prevalent, accounting for 35.3% and 29.5% of the genome, respectively (Supplementary Table S8). This repeat abundance is nearly identical to that (∼77.7%) estimated in the hard-shelled adlay genome (Liu et al., 2019). In addition, this is very similar to that (∼76%) estimated using low-coverage NGS data in the *C. lacryma-jobi* (Cai et al., 2014) and that (∼75%) in the wild adlay (*Coix aquatica*) genome (Guo et al., 2019). In contrast, we detect repeats at a higher abundance than was identified in the sorghum genome (∼62%) (Paterson et al., 2009) and a lower amount than in the maize genome (∼85%) (Schnable et al., 2009). On the other hand, the high proportion of LTR-retrotransposons in the soft-shelled adlay genome is very similar to the repeat compositions found in other *Coix* species (Liu et al., 2019; Guo et al., 2019), sorghum (Paterson et al., 2009), and maize (Schnable et al., 2009) as well as other plant genomes (Kim et al., 2018; Zhang et al., 2019).

We further generated a total of 67.8 Gb of transcriptome data from six tissues, including leaves, roots, flowers, stems, and seeds, which were used as evidence for gene prediction (Supplementary Table S2). Through our evidence-based annotation pipeline (Supplementary Figure S2), a total of 39,574 protein-coding genes (soft-shelled adlay gene set ver. 1.2) were predicted. These have an average length of 1,208 bp and account for 3.7% of the genome sequence (Table 1). Features of this gene set are also similar to those of other plant species, including other *Coix* species, sorghum, maize, foxtail millet, and rice (Supplementary Table S9).

Functional annotation revealed that 84.9% of soft-shelled adlay genes display high sequence similarity with known sequences deposited in GenBank; 59.6% have known conserved domains, and 53.8% can be assigned to at least one GO term. In addition, more than 62.2% of gene products show high similarity with genes in sorghum, maize, rice, and *A. thaliana* at the amino acid level. In total, 33,832 (85.5%) genes from our draft genome were functionally annotated by at least one database (Supplementary Table S10).

### Comparative Analysis and Evolution of the Soft-Shelled Adlay Genome

**FIGURE 1:**
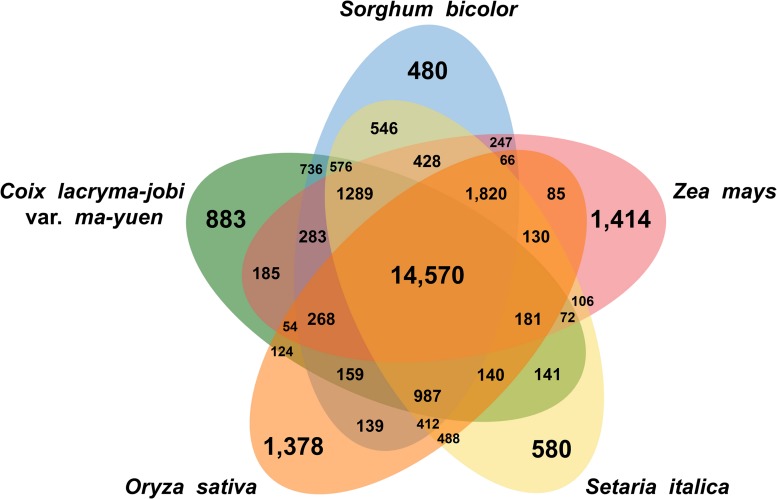
Shared and unique gene clusters in soft-shelled adlay cultivar Johyun and related species. Venn diagram illustrating the number of shared and unique gene clusters in soft-shelled adlay (*C. lacryma-jobi* var. *ma-yuen*), sorghum (*Sorghum bicolor*), maize (*Zea mays)*, foxtail millet (*Setaria italica*), and rice (*Oryza sativa*) was drawn using the OrthoVenn2 web tool, with plant group parameters and an *E*-value cutoff of 1e-5.

Gene clustering analysis based on similarity to known protein sequences from related species revealed that the soft-shelled adlay gene products group into 14,570 gene clusters with shared genes from sorghum, maize, foxtail millet, and rice, as well as 883 clusters with genes unique to soft-shelled adlay (Figure 1). GO enrichment analysis of the shared clusters identified the abundant GO terms, such as Biological Process GO terms related to RNA modification, defense response, response to osmotic stress, recognition of pollen, and the plant-type hypersensitive response, as well as Molecular Function GO terms related to oxidoreductase activity, nutrient reservoir activity, and transferase activity. In contrast, Biological Process GO terms related to DNA integration and DNA recombination were most abundant for the clusters containing genes unique to soft-shelled adlay (Supplementary Table S11).

Additional gene clustering analysis was performed with gene sequences of two other *Coix* species, hard-shelled adlay and wild adlay, whose genomes were most recently reported (Guo et al., 2019; Liu et al., 2019), revealing that 604, 3,859 and 610 gene clusters are unique to soft-shelled adlay, hard-shelled adlay, and wild adlay, respectively, and 19,693 gene clusters are shared among the three *Coix* species (Supplementary Figure S3). GO enrichment analyses revealed that Biological Process GO terms related to DNA integration, DNA recombination, response to red or far red light, transposition, RNA-mediated, and telomere maintenance were abundant in the unique gene clusters of soft-shelled adlay whereas no abundant GO term was found in the unique gene clusters of hard-shelled adlay. Abundant GO terms found in the unique gene clusters of wild adlay were similar to those of soft-shelled adlay (Supplementary Table S11). Taken together, this implies that the three *Coix* genomes have their own unique features. Therefore, these multiple reference genomes for *Coix* species will be valuable for further comparative genomics and molecular breeding for desirable traits such as shell-thickness and stress resistance, as demonstrated in many studies with multiple reference genomes of crops (Zhang et al., 2014; Kim et al., 2017; Lee et al., 2017; Griesmann et al., 2018; Chang et al., 2019).

Conserved domain searches were then performed to compare the domains found in soft-shelled adlay gene products to those present in proteins from other plant species. We identified 5,637 domains in the predicted soft-shelled adlay gene products. Of these, 17 were found to be over-represented in soft-shelled adlay relative to sorghum or other species, based on a differential abundance ≥ 2-fold and *P*-value < 0.05. Among over-represented domains, conserved domains for Ribonuclease H1 (InterPro Entry ID IPR011320), Aspartic peptidase domain (IPR019103, IPR021109), ArgJ-like domain (IPR016117), Chromo-like domain superfamily (IPR016197), PBS lyase HEAT-like repeat (IPR004155), No apical meristem-associated (IPR029466), and CCHC-type Zinc finger (IPR001878 and IPR036875) were over-represented compared to other examined species, including sorghum and maize (Supplementary Table S12). In addition, 1,655 TFs, 422 TRs, and 1,264 PK genes were identified, which account for 4.18%, 1.07%, and 3.19%, respectively, of the total predicted genes in soft-shelled adlay. The proportions of these predicted gene products are also similar to those found in other examined plant species (Supplementary Tables S13–S15).

**FIGURE 2:**
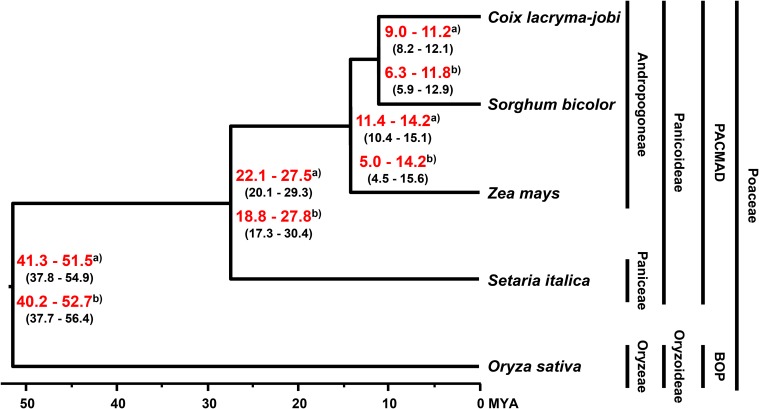
Evolutionary history of soft-shelled adlay. Phylogeny and divergence time were determined by BEAST2 with strict and relaxed clock models, using single-copy orthologous genes in soft-shelled adlay (*C. lacryma-jobi* var. *ma-yuen*), sorghum (*Sorghum bicolor*), maize (*Zea mays)*, foxtail millet (*Setaria italica*), and rice (*Oryza sativa*). The tree was visualized by FigTree. Estimated divergence times were calculated using divergence times from the TimeTree database estimated for the split between foxtail millet-maize and rice (95% confidence interval [CI]: 42–52 MYA) and between maize and sorghum (CI: 9.39–12.47 MYA). The estimated minimum and maximum times are shown within parentheses, and the range of estimated median divergence time are depicted in red bold letters at branching points between species. ^a)^ Divergence time estimated with a strict clock model. ^b)^ Divergence time estimated with a relaxed clock model (Supplementary Figure S5).

The divergence time for soft-shelled adlay within the Poaceae family was estimated by BEAST2 with GTR substitution and a strict clock model, using 630 single-copy genes from soft-shelled adlay and their orthologs from four monocots (sorghum, maize, foxtail millet, and rice). From these data, soft-shelled adlay and sorghum were estimated to have diverged from a common ancestor 9.0–11.2 MYA (median value), and after divergence from maize, which was estimated to have occurred 11.4–14.2 MYA (Figure 2). This estimated divergence time between soft-shelled adlay and sorghum is in agreement with the divergence times previously estimated using *bz* orthologous regions (9.32 MYA, Wang and Dooner, 2012) and the genome of hard-shelled adlay (∼10.4 MYA, Liu et al., 2019). In addition, this is also within the range of divergence times predicted from the TimeTree database for the split between *C. lacryma-jobi* and sorghum (CI: 5.5–16.1 MYA) (Supplementary Figure S4). In addition, divergence time was also estimated with a relaxed clock model and the estimated divergence time (6.3–11.8 MYA) between soft-shelled adlay and sorghum is also within the divergence time range from the TimeTree database (Figure 2 and Supplementary Figures S4, S5).

### Differentially Expressed Genes in Seeds of Soft-Shelled Adlay

**FIGURE 3:**
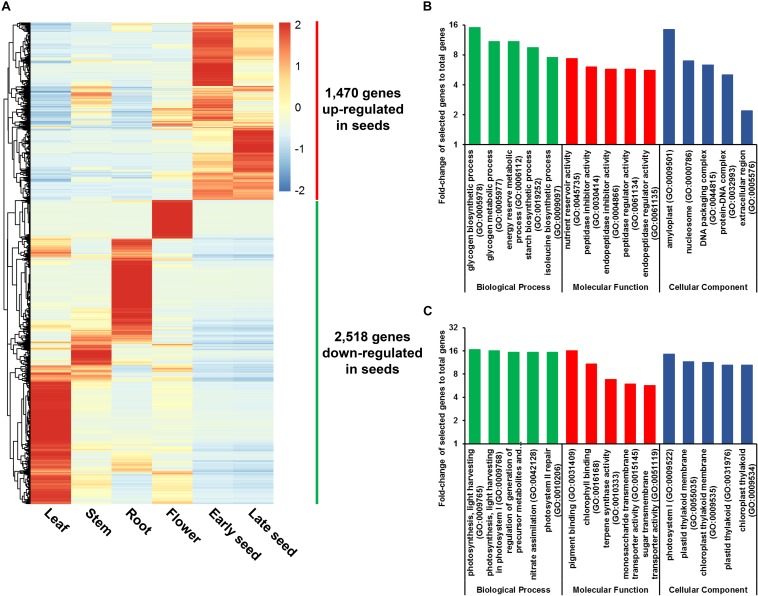
Genes differentially expressed (DE) in seeds. **(A)** Expression patterns for 3,988 DE genes in seeds, which were identified by DESeq, using RNA-Seq data from seeds and other plant tissue samples. Expression values (FPKM) were scaled per row (i.e., per gene) to visualize gene expression peaks among the different tissues, and the heatmap was generated using the R-package pheatmap. Leaves, stems, roots, and flowers, as well as early seeds sampled from 98-day-old plants and late seeds sampled from 159-day-old plants were used in this study. Gene names are omitted. **(B)** Five most abundant Gene Ontology (GO) terms identified in genes up-regulated in seeds. **(C)** Five most abundant GO terms identified in genes down-regulated in seeds. Abundant GO terms were identified by GO enrichment analysis using the following cutoffs: fold-change ≥ 2, *P*-value < 0.01, and false-discovery rate < 0.01. Detailed information is shown in Supplementary Tables S16, S17.

A total of 3,988 genes were found to be DE in seeds relative to other plant tissues, with significant expression changes ≥ 2-fold at adjusted *P*-value < 0.05. Among these DE genes, 1,470 were found to be up-regulated, and 2,518 were found to be down-regulated in seeds, respectively (Figure 3A and Supplementary Table S16). GO enrichment analysis revealed that GO terms related to carbohydrate and protein metabolism, such as glycogen biosynthetic process (GO:0005978) and nutrient reservoir activity (GO:0045735), are abundant among the up-regulated genes, whereas GO terms related to photosynthesis, such as photosynthesis and light harvesting (GO:0009765), are most prevalent for down-regulated genes (Figures 3B,C and Supplementary Table S17). These results provide a possible explanation for the high starch and protein content found in soft-shelled adlay seeds.

In addition, a total of 317 genes were identified to encode regulatory genes including 289 TF and 28 TR genes, among the DE genes in seeds. Of those, 97 TF genes were highly expressed in seeds, which included several important TF families such as AP2/ERF (11), bHLH (9), bZIP (7), MYB (7), and NAC (7) (Supplementary Table S16 and Supplementary Figure S6). These regulatory genes will provide a basis for study on seed development and for molecular breeding of high-quality seed production.

### Storage Protein Genes in Soft-Shelled Adlay

Based on the observation that a GO term related to storage proteins, nutrient reservoir activity (GO:0045735), was detected as the most abundant among Molecular Function GO terms for up-regulated genes in seeds (Figure 3B), gene products assigned to this GO term were investigated to identify additional storage protein-encoding genes in the soft-shelled adlay genome. From these analyses, we identified a total of 76 genes predicted to encode storage proteins, including 57 cupin superfamily genes, 18 coixin genes, and one glutelin gene (Supplementary Table S18). Among these genes, 50 are tandemly arranged in the genome sequence of soft-shelled adlay and form 13 gene clusters. For the 18 coixin genes, 13 are tandemly arranged and form five gene clusters, as reported in previous studies (Ottoboni et al., 1993; Meng et al., 2010; Zhou et al., 2010). Notably, the total number (76) of predicted storage protein-encoding genes in soft-shelled adlay is similar to those in hard-shelled adlay (81), wild adlay (67), sorghum (86), maize (82), foxtail millet (71), and rice (72) (Supplementary Table S19).

**FIGURE 4:**
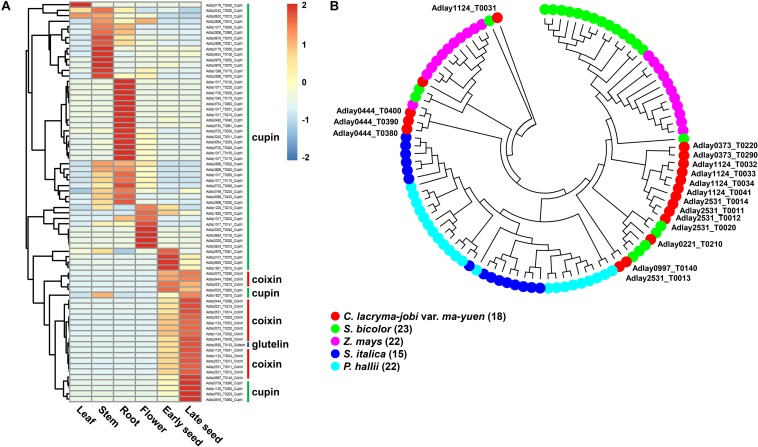
Predicted storage protein-encoding genes in the soft-shelled adlay genome. Seventy-six genes predicted to encode storage proteins (57 cupin superfamily, 18 coixin, and 1 glutelin) were identified, and their expression patterns were analyzed in various tissues. **(A)** Expression patterns of storage protein genes were determined using FPKM values from RNA-Seq data obtained from each tissue sample. Expression values (FPKM) were scaled per row (i.e., per gene) to visualize gene expression peaks among the different tissues, and the heatmap was generated using the R-package pheatmap. Tissue names and gene symbols are shown at the bottom and on the right of the heatmap, respectively. Three genes, Adlay1017_T0202 (cupin superfamily), Adlay1124_T0031 (coixin), and Adlay3240_T0009 (cupin superfamily), were not expressed in any tissue (FPKM = 0) and are therefore omitted from this heatmap. Leaves, stems, roots, and flowers, as well as early seeds sampled from 98-day-old plants and late seeds sampled from 159-day-old plants were used in this study. **(B)** Phylogenetic analysis of 18 coixin proteins containing a zein seed storage protein domain in *C. lacryma-jobi* var. *ma-yuen* ‘Johyun’ (red) and four other plant species: *S. bicolor* (green), *Z. mays* (purple), *S. italica* (blue), and *P. hallii* var. *hallii* (sky blue). The phylogenetic tree was generated using the maximum-likelihood (ML) method with 1,000 bootstraps by MEGA ver. 7.0, after alignment of predicted amino acid sequences by MUSCLE. The bootstrap support values and gene names (except for those from soft-shelled adlay) have been omitted for legibility. Gene numbers are indicated in parentheses after the species names. Detailed information is shown in Supplementary Tables S18–20 and Supplementary Figure S7.

Most storage protein-encoding genes were found to be expressed in some subset of the five tissues examined (leaf, stem, root, flower, and seed), except for three genes, Adlay1017_T0202 (cupin superfamily), Adlay1124_T0031 (coixin), Adlay3240_T0009 (cupin superfamily), which were not detected in any tissue (Figure 4A and Supplementary Table S18). Genes encoding coixin and glutelin proteins are highly expressed in early and late-stage seeds. Among the 57 cupin superfamily genes, most (39 genes) are highly expressed in vegetative tissues, such as the stem and root, whereas the remaining 18 genes show elevated expression in reproductive tissues (flower and seed). This expression pattern may explain why adlay seeds contain large amounts of proteins, particularly coixin proteins (Ottoboni et al., 1990; Lee et al., 2011; Corke et al., 2016; Zhu, 2017).

Most coixin genes display high levels of similarity with reported coixin genes in adlay, and some, such as Gene ID Adlay1124_T0031 and Adlay2531_T0012, are newly identified in this study (Supplementary Table S20). Phylogenetic analysis of predicted proteins sequences reveals that coixin genes from soft-shelled adlay group more closely with those from sorghum (kafirin) and maize (zein) in the Andropogoneae tribe relative to those from foxtail millet and Hall’s panicgrass (*Panicum hallii* var. *hallii*) in the Paniceae tribe (Figure 4B). This grouping pattern is consistent with those reported in previous studies of coixin genes (Ottoboni et al., 1993; Zhou et al., 2010).

### Genes Involved in BX Biosynthesis

**FIGURE 5:**
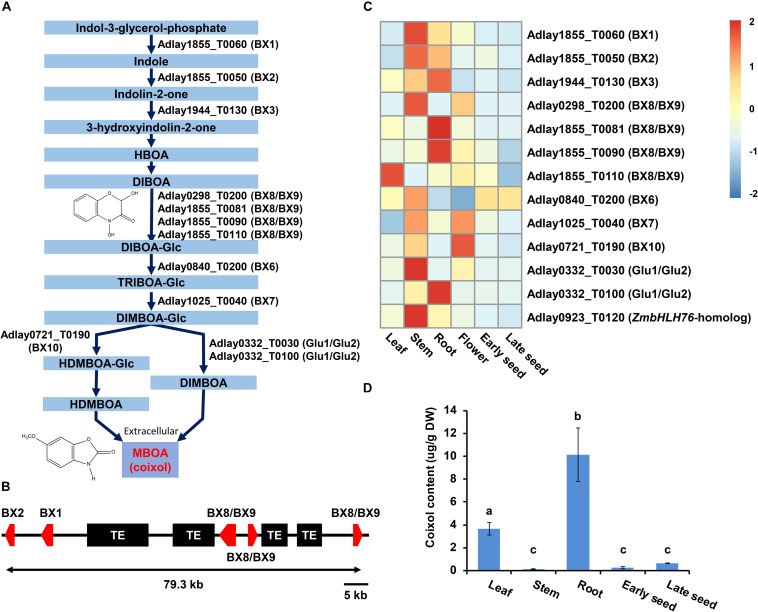
Predicted BX biosynthesis genes in the soft-shelled adlay genome. A total of 13 genes predicted to encode proteins involved in BX biosynthesis were identified, and their expression patterns were analyzed in various tissues. **(A)** Diagram of the BX biosynthesis pathway showing the 12 genes assigned to this pathway (KEGG Entry map00402, https://www.genome.jp/dbget-bin/www_bget?map00402) (Cotton et al., 2019) based on functional annotation information. HBOA, 2-hydroxy-1,4-benzoxazin-3-one; DIBOA, 2,4-dihydroxy-1,4-benzoxazin-3-one; TRIBOA, 2,4,7-trihydroxy-2H-1,4-benzoxazin-3(4H)-one; DIMBOA, 2,4-dihydroxy-7-methoxy-1,4-benzoxazin-3-one; HDMBOA, 2-hydroxy-4,7-dimethoxy-1,4-benzoxazin-3-one; MBOA (coixol), 6-methoxy-benzoxazolin-2-one; Glc, glucoside. **(B)** Schematic diagram of the BX gene cluster. Five genes, including Adlay1855_T0050 (BX2), Adlay1855_T0060 (BX1), Adlay1855_T0081 (BX8/BX9), Adlay1855_T0090 (BX8/BX9), and Adlay1855_T0110 (BX8/BX9) are tandemly located on a 79.3-kb region of Scaffold ID Adlay1855. Red box, BX gene; Black box, transposable element (TE). **(C)** Expression patterns of BX biosynthesis genes and regulators were determined using FPKM values from RNA-Seq data obtained from each tissue sample. Expression values (FPKM) were scaled per row (i.e., per gene) to visualize gene expression peaks among the different tissues, and the heatmap was generated using the R-package pheatmap. Tissue names and gene symbols are shown at the bottom and on the right of the heatmap, respectively. Leaves, stems, roots, and flowers, as well as early seeds sampled from 98-day-old plants and late seeds sampled from 159-day-old plants were used in this study. Detailed information is shown in Supplementary Table S21. **(D)** MBOA (coixol) content in five tissues of soft-shelled adlay, not including flowers. Bar indicates mean and standard deviation of at least two independent biological samples. Different letters indicate significant differences between groups [one-way ANOVA followed by Duncan’s least significant range (LSR) test, *P*-value < 0.05].

A total of 13 candidate genes was identified as likely to be involved in the BX biosynthesis pathway in soft-shelled adlay, promoting the conversion of indole-3-glycerol-phosphate to 2,4-dihydroxy-7-methoxy-1,4-benzoxazin-3-one (DIMBOA) (Figure 5A, Supplementary Table S21, and Supplementary Figures S8–S14), which is then known to be spontaneously degraded to 6-methoxy-1,3-benzoxazol-2-one (MBOA, coixol) in soft-shelled adlay. Among these 13 genes, five (Adlay1855_T0050 [BX2], Adlay1855_T0060 [BX1], Adlay1855_T0081 [BX8/BX9], Adlay1855_T0090 [BX8/BX9], and Adlay1855_T0110 [BX8/BX9] form a tandem gene cluster on a 79.3-kb region of the soft-shelled adlay genome (Figure 5B) that is collinear with the BX gene cluster reported in maize genome (Supplementary Figure S15) (Zheng et al., 2015). Furthermore, the five BX gene cluster shows complete microsynteny among the three *Coix* species (Supplementary Figure S16). In addition to BX genes, a bHLH TF gene (Adlay0923_T0120) was also identified as a homolog to the maize positive regulator, ZmbHLH76, for BX biosynthesis genes (Gao et al., 2019).

Expression analysis revealed that most BX and bHLH genes are highly expressed in stem and root tissues relative to all six tissues examined, although some were found to be highly expressed in leaf and flower tissues. Conversely, all predicted BX genes display weak or undetectable expression in seeds (Figure 5C and Supplementary Table S21). These BX gene expression patterns were very similar to those in maize (Supplementary Figure S17).

These BX gene expression patterns are likely to contribute to the high accumulation of coixol in roots and low coixol levels in seeds (Figure 5D) (Otsuka et al., 1988; Choi et al., 2017).

## Conclusion

In this study, we report the draft genome sequence and genomic characterization of soft-shelled adlay, *C. lacryma-jobi* var. *ma-yuen*. We identified genes that are likely to contribute to the unique phenotype of this crop at the molecular level, including storage protein genes and BX biosynthesis genes. To our knowledge, this is the first report of a draft genome sequence for soft-shelled adlay species, and our data will therefore be a valuable resource for both molecular breeding and pharmacological studies of this species. In addition, our data contribute to the total genomic resources available for Poaceae species, thereby enhancing our understanding of evolution within the Poaceae family.

## Data Availability Statement

The datasets generated for this study can be found in the NCBI (Bioproject accession PRJNA573577, https://www.ncbi.nlm.nih.gov/bioproject/PRJNA573577). This Whole Genome Shotgun project has been deposited at DDBJ/ENA/GenBank under the accession number WLYU00000000. The version described in this paper is version WLYU01000000. In addition, the genome assembly and annotation information is also available in *Coix lacryma-jobi* Genome DB (http://phyzen.iptime.org/adlay/index.php). The list of software, parameters and database resources used in this study is shown in Supplementary Table S22.

## Author Contributions

S-HK, S-CL, KK, T-JO, and C-KK conceived of this genome project and coordinated research activities. S-JK prepared plant materials and sequencing data. B-SC, N-HK, HL, SL, HK, MS, H-WK, KN, and S-CL assembled and annotated the genome sequence and performed bioinformatics analyses. BK, T-JO, and S-CL analyzed the genes involved in BX biosynthesis. S-JK provided advice and associated information. S-CL, BK, T-JO, SL, and C-KK wrote the manuscript. All authors have read and approved the final manuscript.

## Conflict of Interest

B-SC, HL, N-HK, HK, MS, H-WK, KN, KK, and S-CL were employed by the company Phyzen Co. SL was employed by the company DNA Link, Inc. The remaining authors declare that the research was conducted in the absence of any commercial or financial relationships that could be construed as a potential conflict of interest.

## Footnotes

1 http://nabic.rda.go.kr/nagp/

2 http://nongup.gg.go.kr/

3 http://www.clcbio.com

4 https://software.broadinstitute.org/gatk

5 http://www.repeatmasker.org/RepeatModeler/

6 http://www.repeatmasker.org

7 https://www.girinst.org/repbase/

8 http://plants.ensembl.org/Zea_mays/Info/Index

9 https://phytozome.jgi.doe.gov/pz/portal.html#!info?alias=Org_Sbicolor

10 https://rapdb.dna.affrc.go.jp/index.html

11 https://www.arabidopsis.org/

12 https://www.ncbi.nlm.nih.gov/protein/?term=txid4505[Organism:exp]

13 https://www.genome.jp/tools/kaas/

14 http://tree.bio.ed.ac.uk/software/figtree/

15 https://CRAN.R-project.org/package=pheatmap

16 https://www.ebi.ac.uk/interpro/entry/IPR002530
