# Supplementary material for: Genome Assembly and Annotation of Soft-Shelled Adlay (Coix lacryma-jobi Variety ma-yuen), a Cereal and Medicinal Crop in the Poaceae Family
Source: Front Plant Sci. 2020 May 18;11:630. doi: 10.3389/fpls.2020.00630 (PMC7247446; doi:10.3389/fpls.2020.00630)
Supplement: Supplementary file 1 [file Table_1.docx]

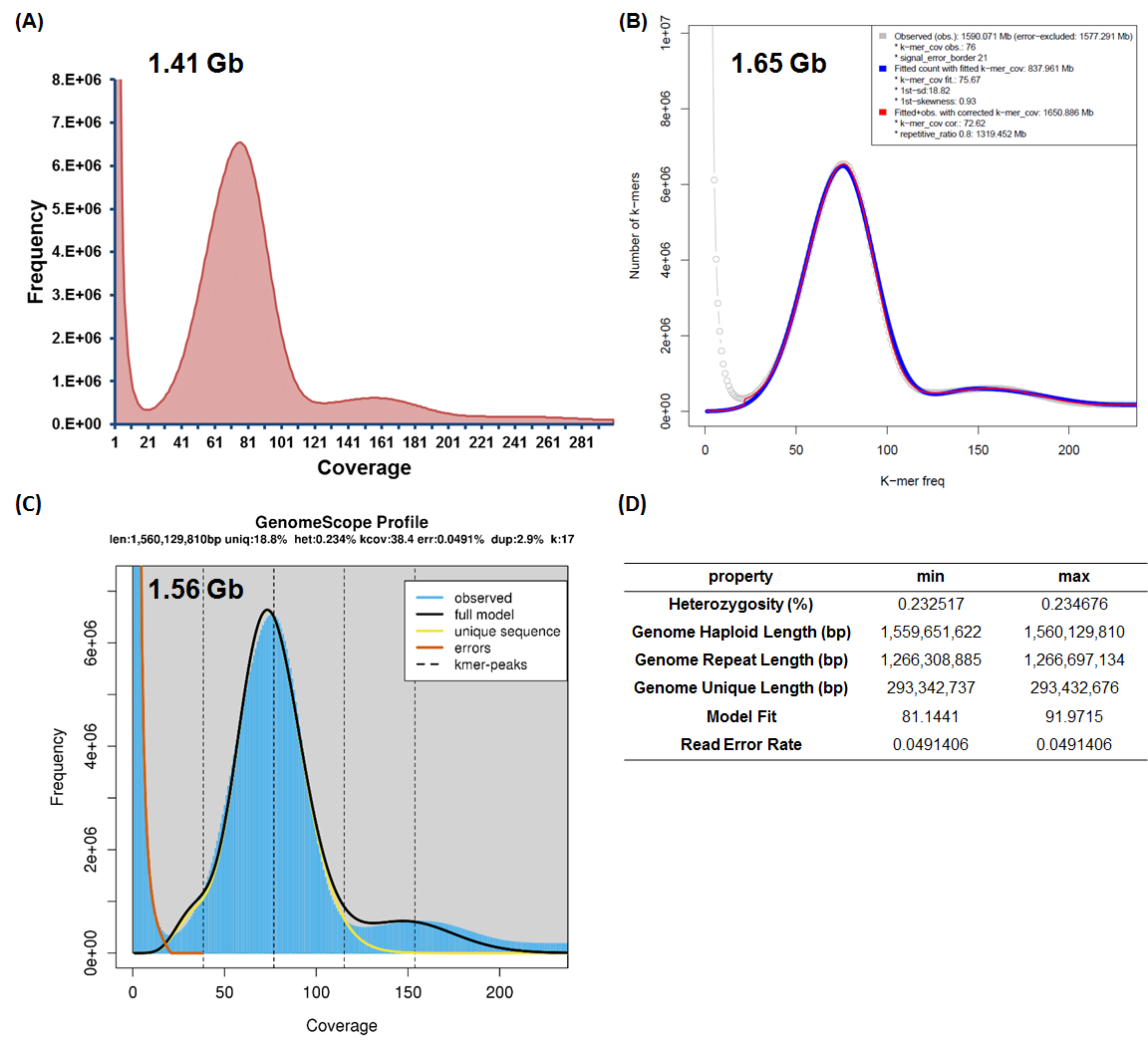


**Supplementary Figure S1.** Genome size estimation of soft-shelled adlay cultivar. Johyun. Genome size was estimated by *k*-mer frequency analyses of trimmed Illumina paired-end (PE) data using **(A)** JELLYFISH ver. 2.0 (Marçais and Kingsford, 2011) and a formula (Genome size = *k*-mer coverage/mean *k*-mer depth), **(B)** findGSE ver. 1.0 (Sun et al., 2018b), and **(C, D)** GenomeScope (Vurture et al., 2017), with an optimal *k*-mer value of 17. Based on these three analyses, the genome size of soft-shelled adlay cv. Johyun was estimated to be 1.41, 1.56, and 1.65 Gb, respectively. From these results, the median value of 1.56 Gb was selected and used in this study; this is consistent with the previously reported genome sizes of adlay of 1.57 Gb (Plant DNA C-values Database, [https://cvalues.science.kew.org/](https://cvalues.science.kew.org/))) but slightly smaller than 1.68 Gb estimated using low-coverage NGS data of adlay (Cai et al., 2014) and 1.80 Gb of hard-shelled adlay (Liu et al., 2019). In addition, analysis using SGA preqc module (<https://github.com/jts/sga/wiki/preqc>) estimated genome size of 1.49 Gb for soft-shelled adlay cultivar Johyun.


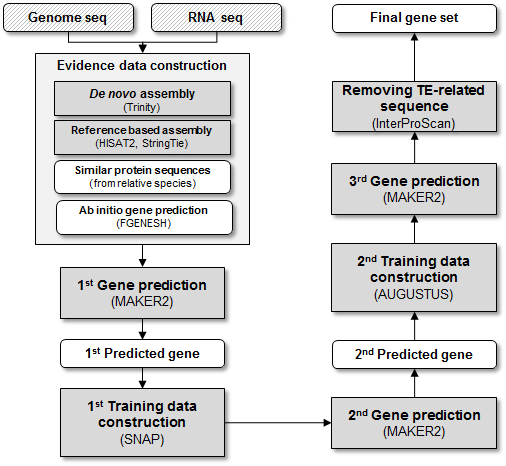


**Supplementary Figure S2.** Annotation pipeline for gene prediction from the draft genome sequence of soft-shelled adlay cultivar Johyun. Trinity ver. 2.1.1, HISAT2 ver. 2.1.0, StringTie ver. 1.3.5, MAKER2 ver. 2.31.8, SNAP ver. 2006-07-28, AUGUSTUS ver. 3.3.2, and InterProScan ver. 5.34-73.0 were used for this analysis with default parameters.


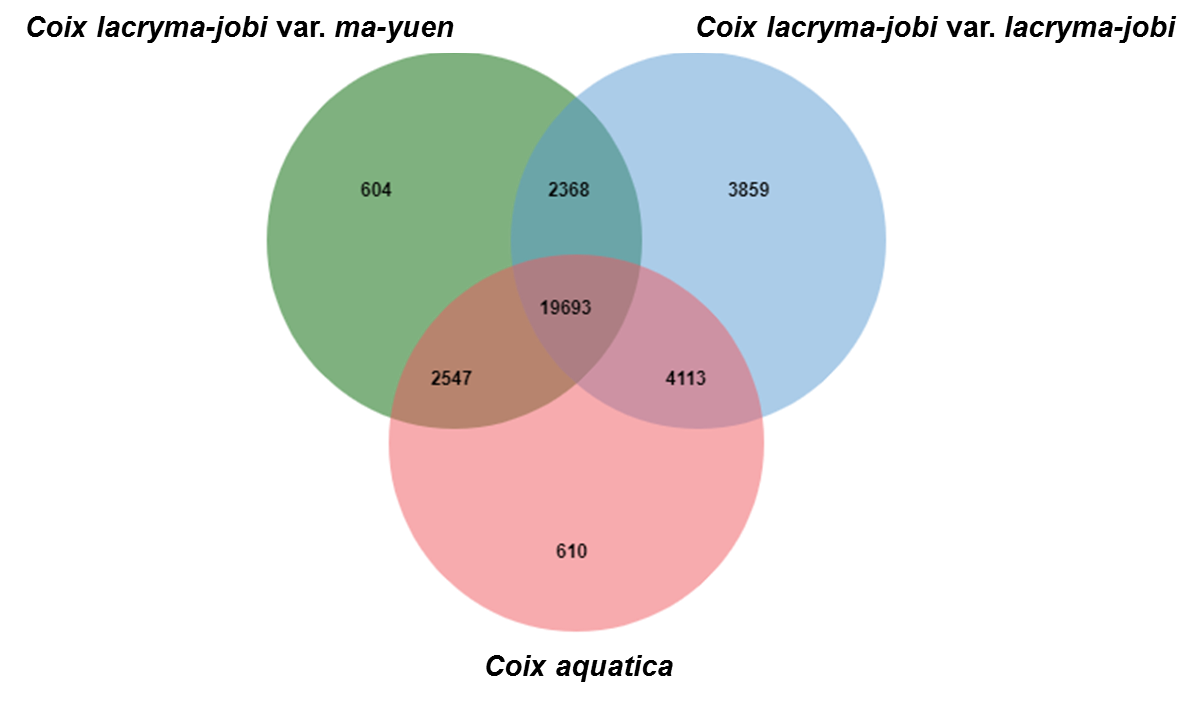


**Supplementary Figure S3**. Shared and unique gene clusters in soft-shelled adlay cultivar Johyun and two other *Coix* species*.* Venn diagram illustrating the number of shared and unique gene clusters in soft-shelled adlay (*Coix lacryma-jobi* var. *ma-yuen* ‘Johyun’) in this study*,* hard-shelled adlay (*Coix lacryma-jobi* var. *lacryma-jobi*) and wild adlay (*Coix aquatica)* was drawn using the OrthoVenn2 web tool (Xu et al., 2019), with plant group parameters and an *E*-value cutoff of 1e-5.


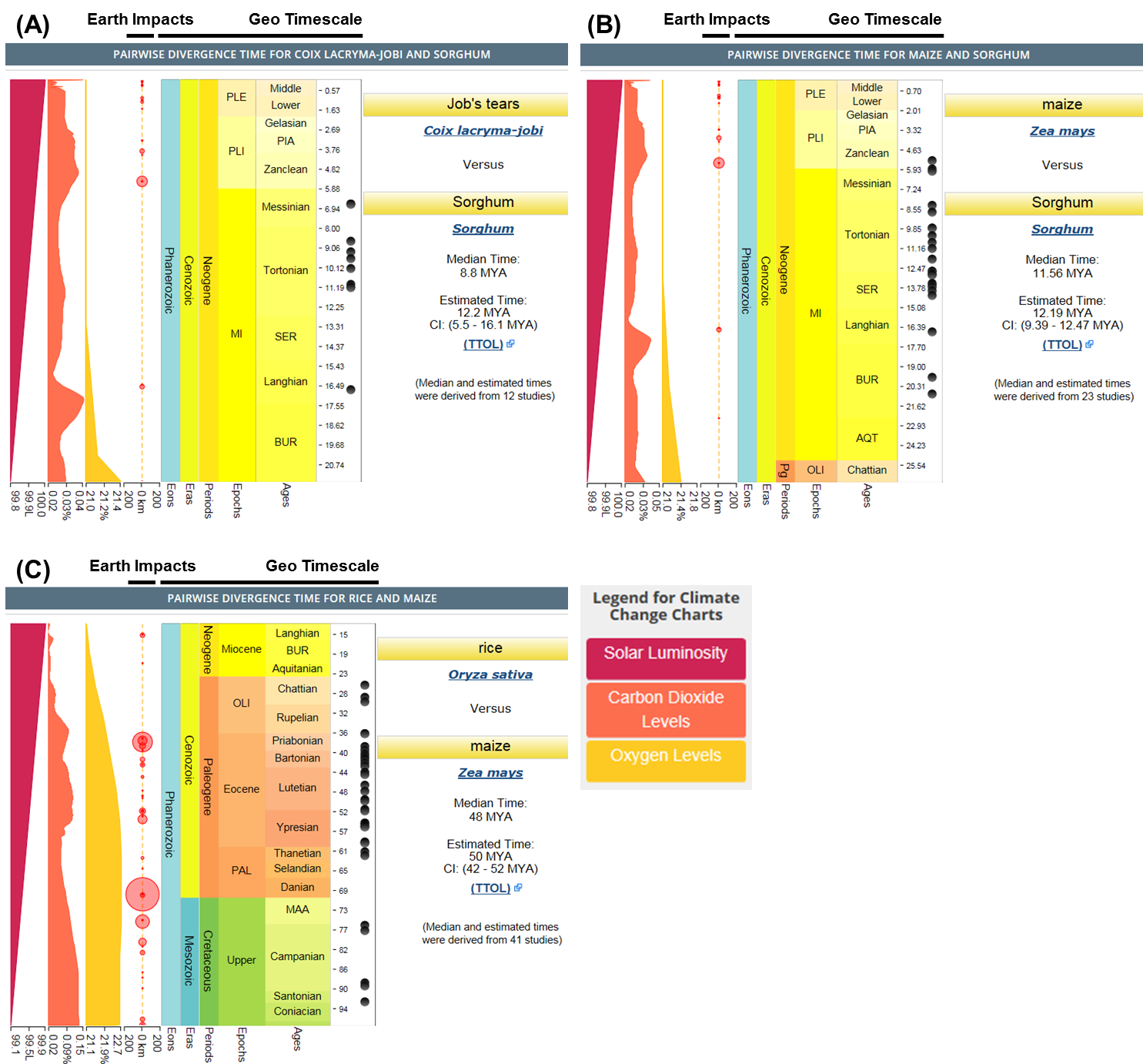


**Supplementary Figure S4**. Pair-wise divergence time estimation for adlay and sorghum **(A)**, maize and sorghum **(B)**, and rice and maize **(C)** provided by the TimeTree database ([http://www.timetree.org](http://www.timetree.org/)). Accessed on 12 March 2020. The divergence time estimation for adlay and sorghum, Cl: 5.5-16.1 MYA; for maize and sorghum, Cl: 9.39-12.47 MYA; for rice and maize, Cl: 42-52 MYA**.**


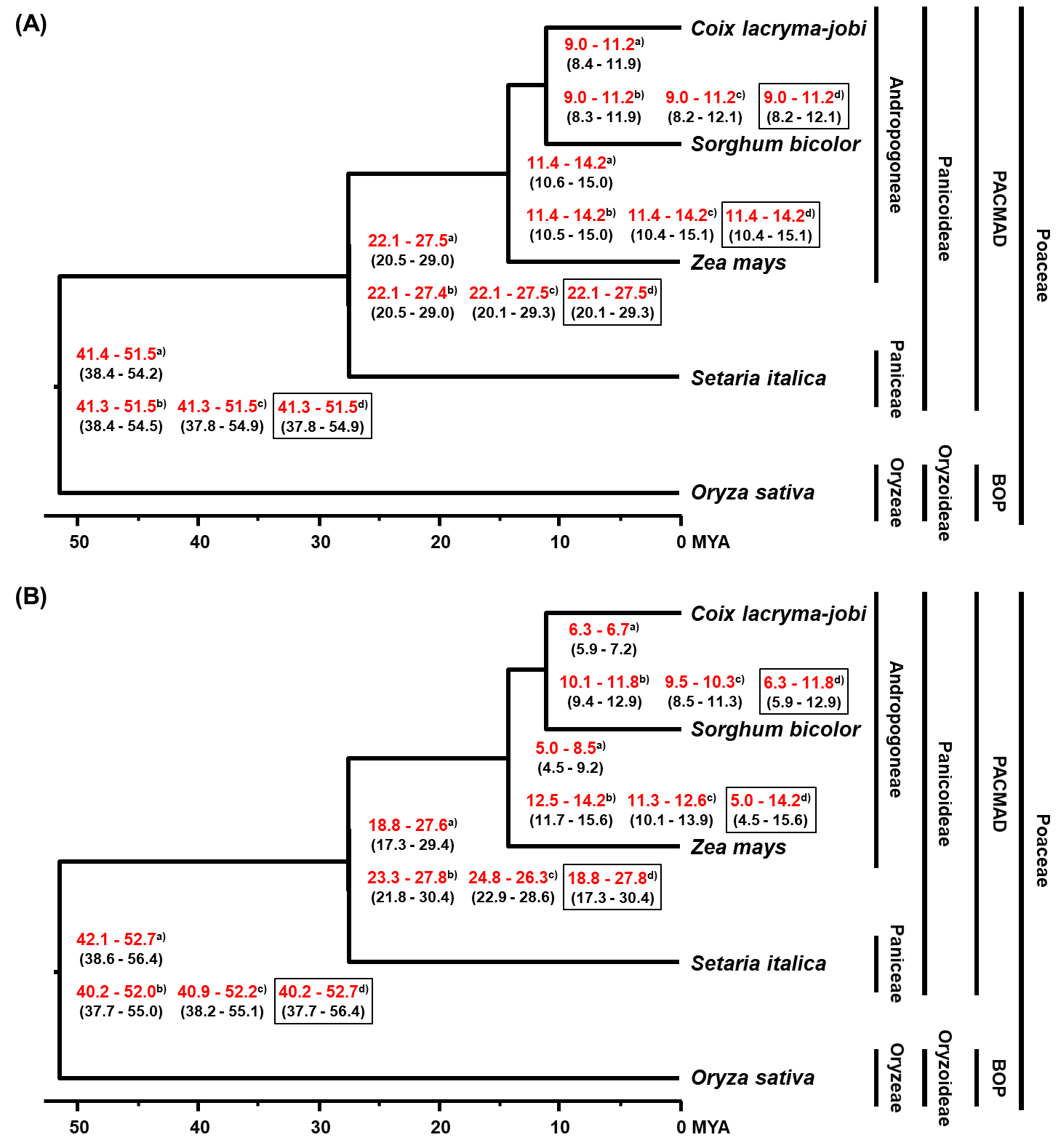


**Supplementary Figure S5.** Evolutionary history of soft-shelled adlay estimated by a strict clock model **(A)** and a relaxed clock model **(B)***.* Phylogeny and divergence time were determined by BEAST2 ver. 2.4.3 (Bouckaert et al., 2014) with GTR substitution and a strict or a relaxed clock model, using single-copy orthologous genes in soft-shelled adlay (*C. lacryma-jobi* var. *ma-yuen* ‘Johyun’)*,* sorghum (*Sorghum bicolor*), maize (*Zea mays)*, foxtail millet (*Setaria italica*) and rice (*Oryza sativa*). The tree was visualized by FigTree ver. 1.4.3 (<http://tree.bio.ed.ac.uk/software/figtree/>). Estimated divergence times were calculated using divergence times from the TimeTree database (Kumar et al., 2017) estimated for the split between foxtail millet-maize and rice (95% confidence interval [CI]: 42-52 MYA) and between maize and sorghum (CI: 9.39-12.47 MYA). The estimated minimum and maximum times are shown within parentheses, and the estimated median divergence times are depicted in red bold letters at branching points between species. ^a), b), c)^ Divergence times estimated three times by BEAST2 with both models. ^d)^ Summarized divergence times of the three estimates.

**Supplementary Figure S6**. Genes encoding transcription factors (TFs, total 289 genes) and transcriptional regulators (TRs, total 28 genes) among differentially expressed (DE) genes in seeds. **(A)** Expression patterns for 97 TF and 14 TR genes among up-regulated genes in seeds. **(B)** Expression patterns for 192 TF and 14 TR genes among down-regulated genes in seeds. Expression values (Fragments per kilobase of transcript per million mapped reads; FPKM) were scaled per row (i.e., per gene) to visualize gene expression peaks among the different tissues, and the heatmap was generated using the R-package pheatmap ver. 1.0.12 (<https://CRAN.R-project.org/package=pheatmap>) with modified parameters (scaling among samples, clustering distance of Euclidean, clustering method of complete). Leaves, stems, roots, and flowers, as well as early seeds sampled from 98-day-old plants and late seeds sampled from 159-day old plants were used in this study. Detailed information is shown in Supplementary Table S16.


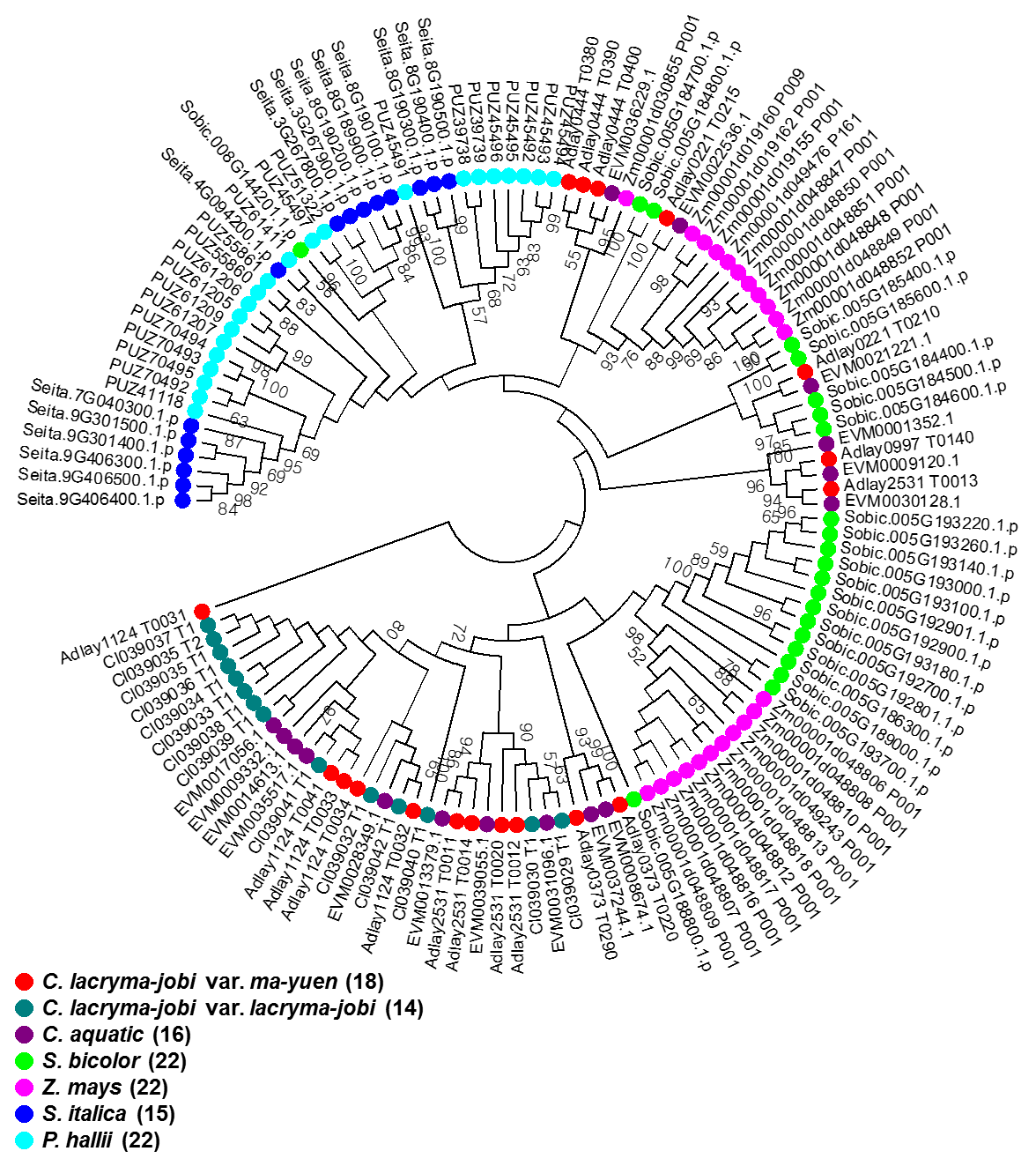


**Supplementary Figure S7.** Detailed phylogenetic tree for 18 coixin proteins containing a zein seed storage protein domain in *C. lacryma-jobi* var. *ma-yuen* ‘Johyun’ (red) and six other plant species. This phylogenetic tree was generated using the maximum likelihood (ML) method with 1,000 bootstraps by MEGA ver. 7.0 (Kumar et al., 2016) with default parameters (JTT substitution model, uniform rates among sites, complete deletion for gap/missing data treatment, NNI ML heuristic method, NJ/BioNJ for initial tree make), after alignment of predicted amino acid sequences by MUSCLE with default parameters (gap open, -2.9; gap extend, 0; hydrophobicity multiplier, 1.2; max iterations, 8; clustering method, UPGMB; min diag length, 24) in MEGA ver. 7.0. Bootstrap support values (≥50%) and gene names are shown. Gene numbers are indicated in parentheses after species names, and detailed information is shown in Supplementary Table S20.


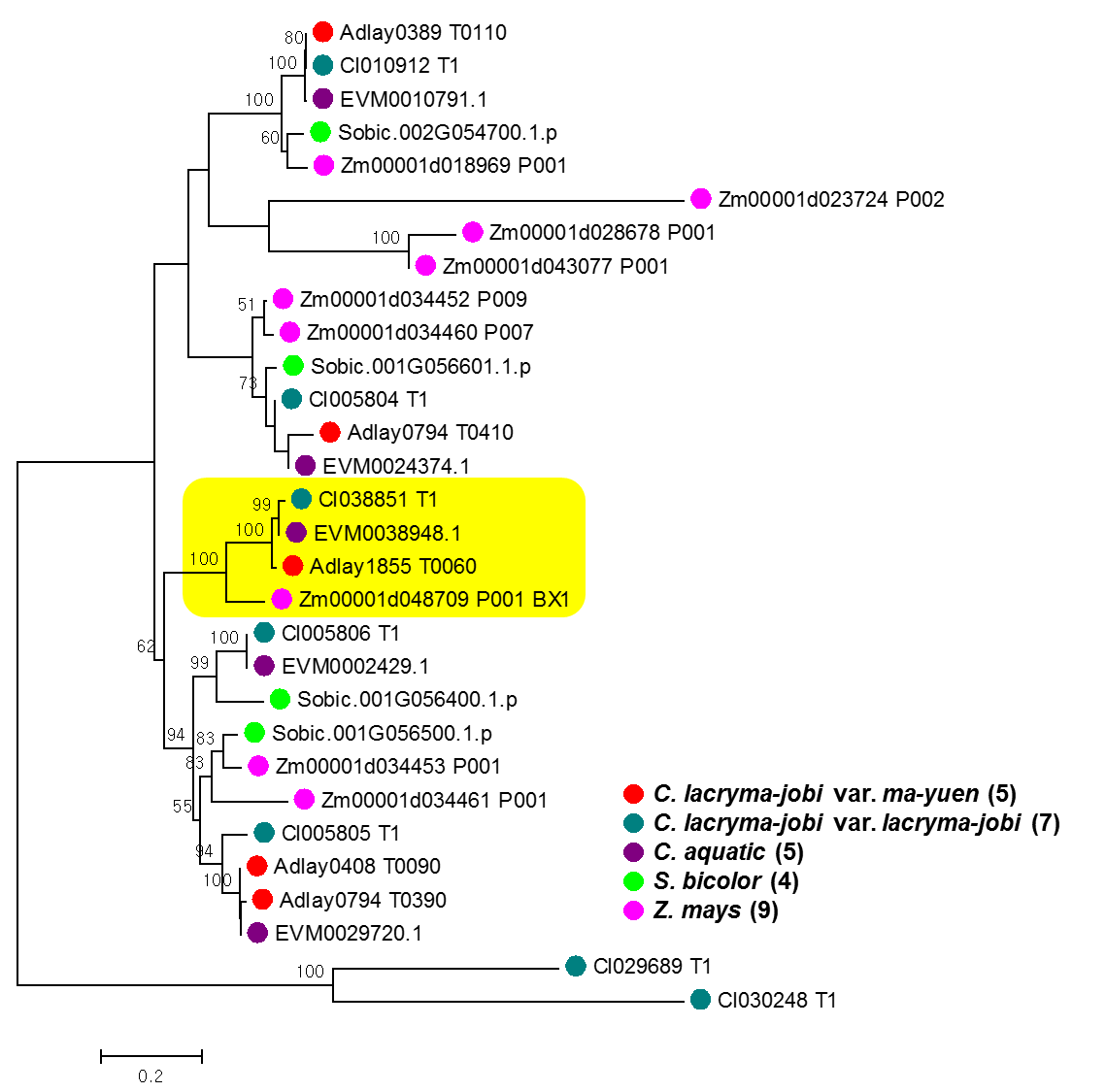


**Supplementary Figure S8.** Phylogenetic tree for tryptophan synthase proteins with conserved tryptophan synthase, alpha chain domain (Interpro entry ID: IPR002028, <https://www.ebi.ac.uk/interpro/entry/InterPro/IPR002028/>). Tree for all tryptophan synthase proteins in *C. lacryma-jobi* var. *ma-yuen* ‘Johyun’ (red), *C. lacryma-jobi* var. *lacryma-jobi* (dark green), *C. aquatica* (dark purple), *S. bicolor* (green)*,* and *Z. mays* (purple) was generated using the Neighbor Joining (NJ) method with 1,000 bootstraps by MEGA ver. 7.0 (Kumar et al., 2016) with default parameters (Poisson substitution model, uniform rates among sites, complete deletion for gap/missing data treatment), after alignment of predicted amino acid sequences by MUSCLE with default parameters (gap open, -2.9; gap extend, 0; hydrophobicity multiplier, 1.2; max iterations, 8; clustering method, UPGMB; min diag length, 24) in MEGA ver. 7.0. Gene numbers are indicated in parentheses after species names. A subtree for proteins closely grouped with *Z. mays* BX1 protein is highlighted in yellow. Bootstrap support values (≥50%) and gene names are shown.


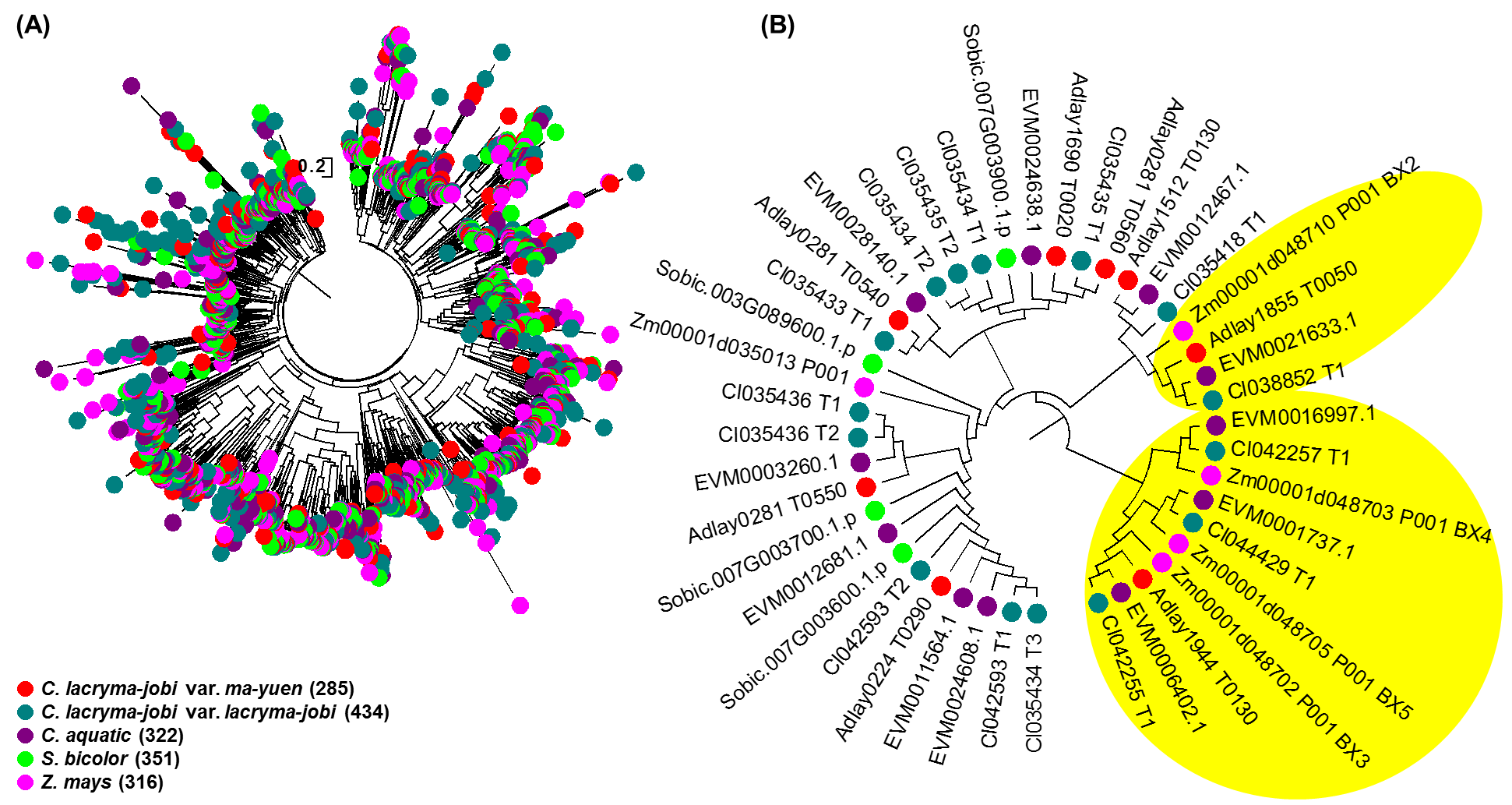


**Supplementary Figure S9.** Phylogenetic tree for cytochrome P450 proteins with conserved cytochrome P450 domain (Interpro entry ID: IPR001128, <https://www.ebi.ac.uk/interpro/entry/InterPro/IPR001128/>). **(A)** Tree for all cytochrome P450 proteins in *C. lacryma-jobi* var. *ma-yuen* ‘Johyun’ (red), *C. lacryma-jobi* var. *lacryma-jobi* (dark green), *C. aquatica* (dark purple), *S. bicolor* (green)*,* and *Z. mays* (purple). Gene numbers are indicated in parentheses after species names. **(B)** Enlarged subtree for proteins closely grouped with *Z. mays* BX2, BX3, BX4, and BX5 proteins. This phylogenetic tree was generated using multiple alignment of predicted amino acid sequences and the Neighbor Joining (NJ) method by MUSCLE ver. 3.8.31 (<http://www.drive5.com/muscle/>) with default parameters and then visualized by MEGA ver. 7.0 (Kumar et al., 2016). Bootstrap support values were not determined here because the NJ tree generated by MUSCLE did not provide the value.


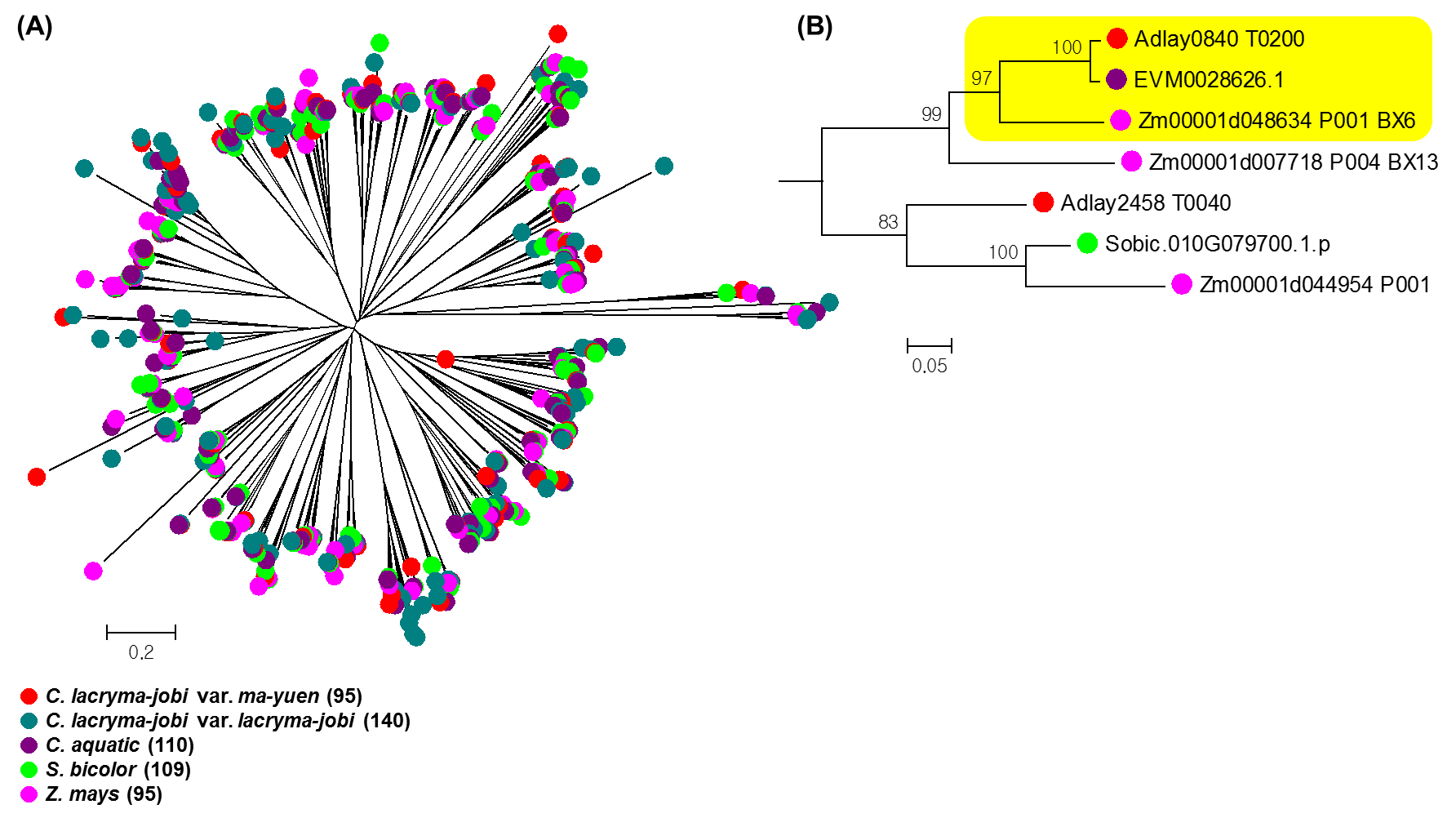


**Supplementary Figure S10.** Phylogenetic tree for oxoglutarate/iron-dependent dioxygenase proteins with conserved oxoglutarate/iron-dependent dioxygenase domain (Interpro entry ID: IPR005123, <https://www.ebi.ac.uk/interpro/entry/InterPro/IPR005123/>). **(A)** Tree for all oxoglutarate/iron-dependent dioxygenase proteins in *C. lacryma-jobi* var. *ma-yuen* ‘Johyun’ (red), *C. lacryma-jobi* var. *lacryma-jobi* (dark green), *C. aquatica* (dark purple), *S. bicolor* (green)*,* and *Z. mays* (purple). Gene numbers are indicated in parentheses after species names. **(B)** Enlarged subtree for proteins closely grouped with *Z. mays* BX6 protein. This phylogenetic tree was generated using the Neighbor Joining (NJ) method with 1,000 bootstraps by MEGA ver. 7.0 (Kumar et al., 2016) with default parameters (Poisson substitution model, uniform rates among sites, complete deletion for gap/missing data treatment), after alignment of predicted amino acid sequences by MUSCLE with default parameters (gap open, -2.9; gap extend, 0; hydrophobicity multiplier, 1.2; max iterations, 8; clustering method, UPGMB; min diag length, 24) in MEGA ver. 7.0. Bootstrap support values (≥50%) and gene names are shown.


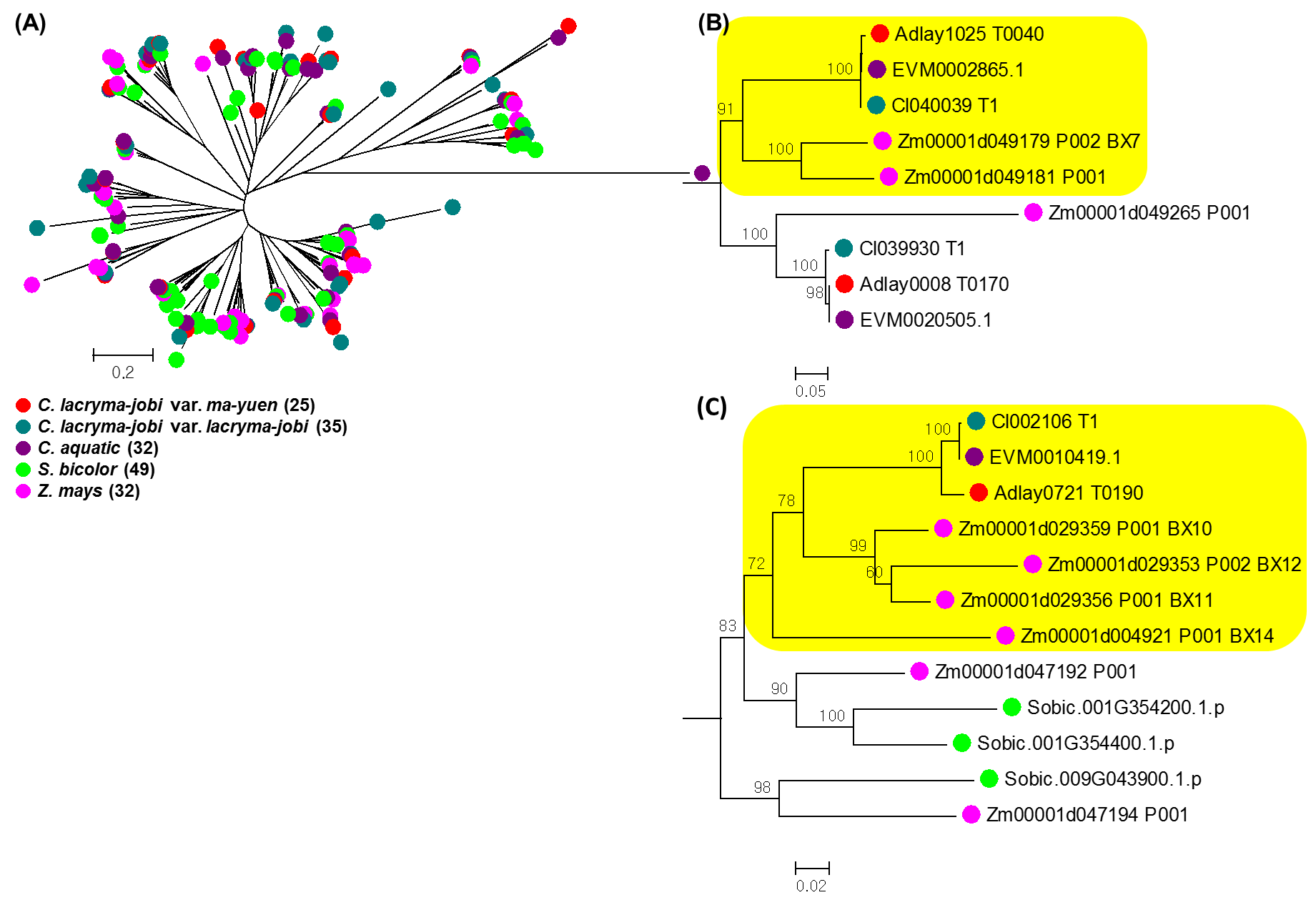


**Supplementary Figure S11.** Phylogenetic tree for O-methyltransferase proteins with conserved O-methyltransferase domain (Interpro entry ID: IPR001077, [https://www.ebi.ac.uk/interpro/entry/InterPro/IPR001077](https://www.ebi.ac.uk/interpro/entry/InterPro/IPR001077/)). **(A)** Tree for all O-methyltransferase proteins in *C. lacryma-jobi* var. *ma-yuen* ‘Johyun’ (red), *C. lacryma-jobi* var. *lacryma-jobi* (dark green), *C. aquatica* (dark purple), *S. bicolor* (green)*,* and *Z. mays* (purple). Gene numbers are indicated in parentheses after species names. **(B)** Enlarged subtree for proteins closely grouped with *Z. mays* BX7 protein. **(C)** Enlarged subtree for proteins closely grouped with *Z. mays* BX10, BX11, BX12, and BX14 proteins. This phylogenetic tree was generated using the Neighbor Joining (NJ) method with 1,000 bootstraps by MEGA ver. 7.0 (Kumar et al., 2016) with default parameters (Poisson substitution model, uniform rates among sites, complete deletion for gap/missing data treatment), after alignment of predicted amino acid sequences by MUSCLE with default parameters (gap open, -2.9; gap extend, 0; hydrophobicity multiplier, 1.2; max iterations, 8; clustering method, UPGMB; min diag length, 24) in MEGA ver. 7.0. Bootstrap support values (≥50%) and gene names are shown.


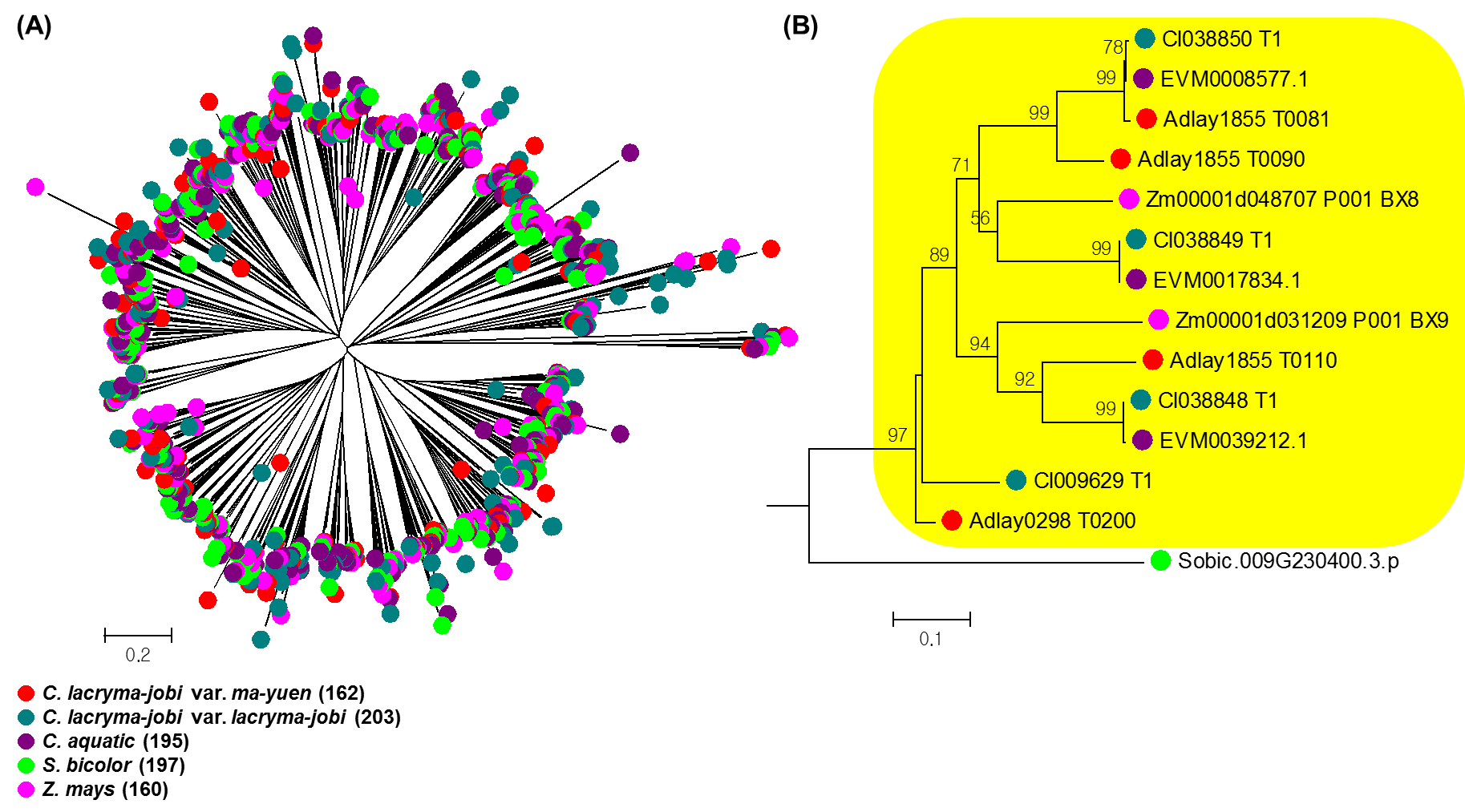


**Supplementary Figure S12.** Phylogenetic tree for UDP-glucosyltransferase proteins with conserved UDP-glucuronosyl and UDP-glucosyl transferase domain (Interpro entry ID: IPR002213, <https://www.ebi.ac.uk/interpro/entry/IPR002213>). **(A)** Tree for all UDP-glucosyltransferase proteins in *C. lacryma-jobi* var. *ma-yuen* ‘Johyun’ (red), *C. lacryma-jobi* var. *lacryma-jobi* (dark green), *C. aquatica* (dark purple), *S. bicolor* (green)*,* and *Z. mays* (purple). Gene numbers are indicated in parentheses after species names. **(B)** Enlarged subtree for proteins closely grouped with *Z. mays* BX8 and BX9 proteins. This phylogenetic tree was generated using the Neighbor Joining (NJ) method with 1,000 bootstraps by MEGA ver. 7.0 (Kumar et al., 2016) with default parameters (Poisson substitution model, uniform rates among sites, complete deletion for gap/missing data treatment), after alignment of predicted amino acid sequences by MUSCLE with default parameters (gap open, -2.9; gap extend, 0; hydrophobicity multiplier, 1.2; max iterations, 8; clustering method, UPGMB; min diag length, 24) in MEGA ver. 7.0. Bootstrap support values (≥50%) and gene names are shown.


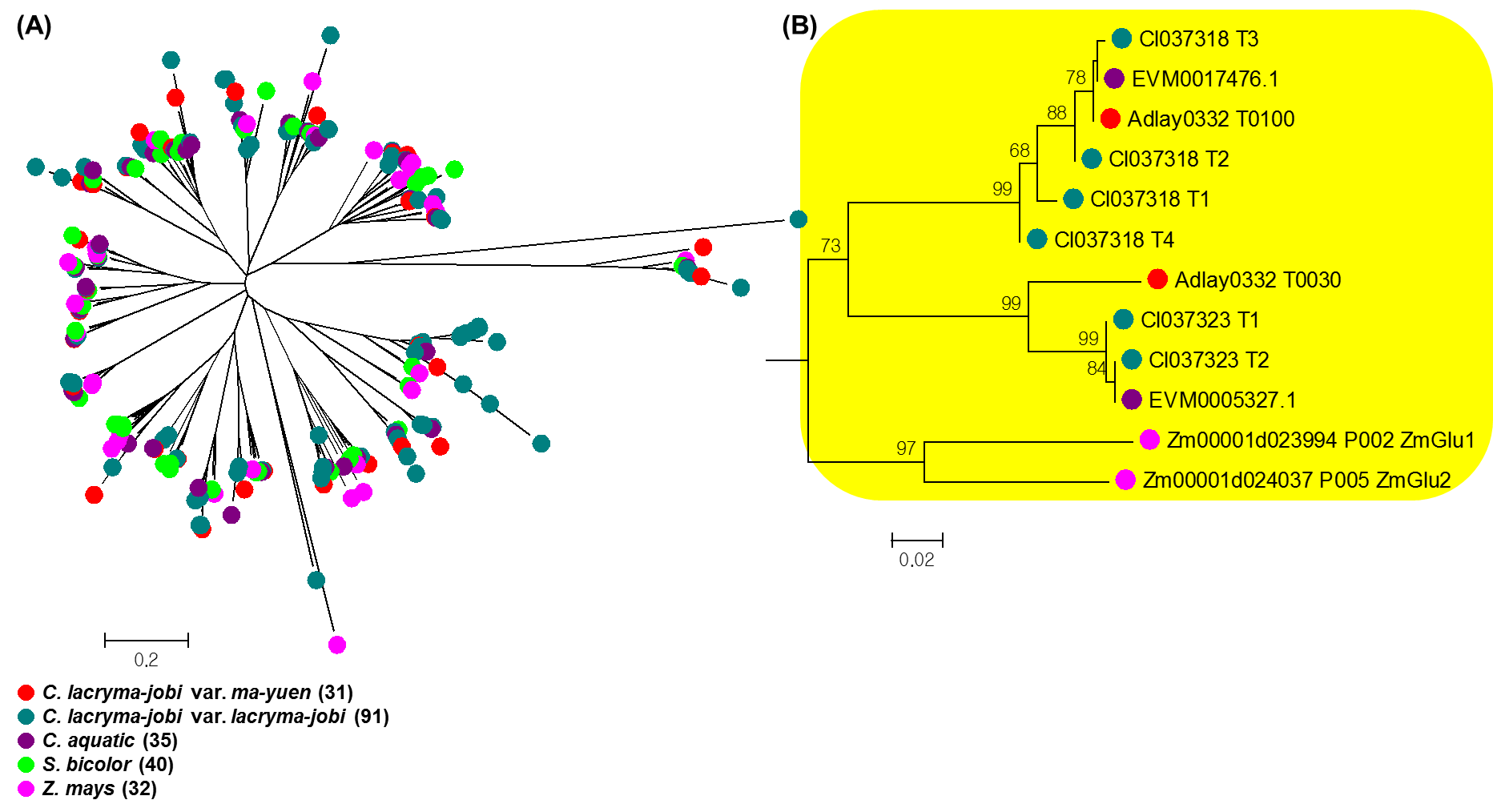


**Supplementary Figure S13.** Phylogenetic tree for glycoside hydrolase proteins with conserved glycoside hydrolase family 1 domain (Interpro entry ID: IPR001360, <https://www.ebi.ac.uk/interpro/entry/InterPro/IPR001360>). **(A)** Tree for all glycoside hydrolase proteins in *C. lacryma-jobi* var. *ma-yuen* ‘Johyun’ (red), *C. lacryma-jobi* var. *lacryma-jobi* (dark green), *C. aquatica* (dark purple), *S. bicolor* (green)*,* and *Z. mays* (purple). Gene numbers are indicated in parentheses after species names. **(B)** Enlarged subtree for proteins closely grouped with *Z. mays* ZmGlu1 and ZmGlu2 proteins. This phylogenetic tree was generated using the Neighbor Joining (NJ) method with 1,000 bootstraps by MEGA ver. 7.0 (Kumar et al., 2016) with default parameters (Poisson substitution model, uniform rates among sites, complete deletion for gap/missing data treatment), after alignment of predicted amino acid sequences by MUSCLE with default parameters (gap open, -2.9; gap extend, 0; hydrophobicity multiplier, 1.2; max iterations, 8; clustering method, UPGMB; min diag length, 24) in MEGA ver. 7.0. Bootstrap support values (≥50%) and gene names are shown.


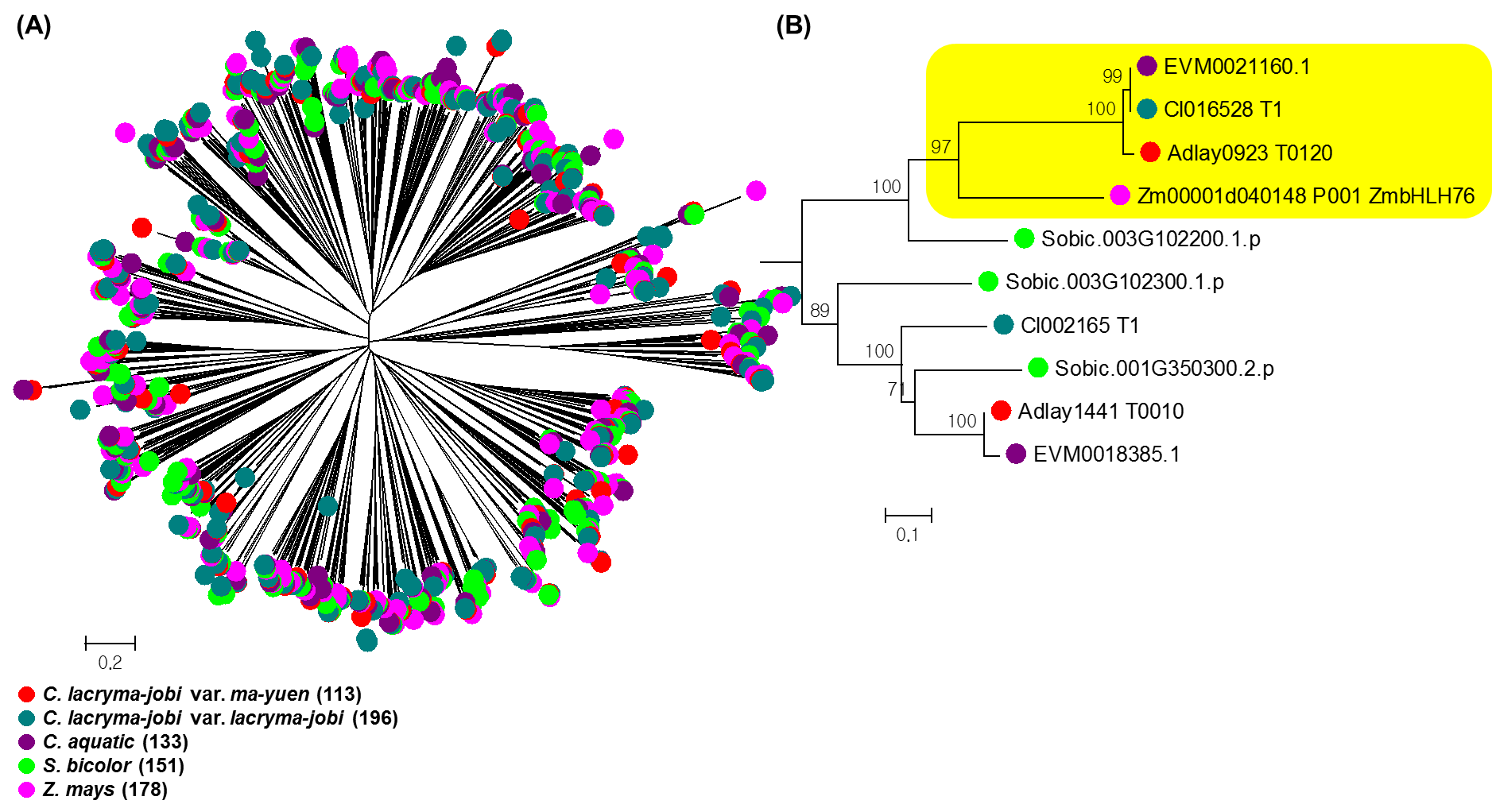


**Supplementary Figure S14.** Phylogenetic tree for basic helix-loop-helix (bHLH) transcription factor proteins. Gene members belonging to the bHLH gene family were identified using iTAK ver. 1.7a stand-alone program (Zheng et al., 2016). **(A)** Tree for all bHLH transcription factor proteins in *C. lacryma-jobi* var. *ma-yuen* ‘Johyun’ (red), *C. lacryma-jobi* var. *lacryma-jobi* (dark green), *C. aquatica* (dark purple), *S. bicolor* (green)*,* and *Z. mays* (purple). Gene numbers are indicated in parentheses after species names. **(B)** Enlarged subtree for proteins closely grouped with *Z. mays* ZmbHLH76 protein. This phylogenetic tree was generated using the Neighbor Joining (NJ) method with 1,000 bootstraps by MEGA ver. 7.0 (Kumar et al., 2016) with default parameters (Poisson substitution model, uniform rates among sites, complete deletion for gap/missing data treatment), after alignment of predicted amino acid sequences by MUSCLE with default parameters (gap open, -2.9; gap extend, 0; hydrophobicity multiplier, 1.2; max iterations, 8; clustering method, UPGMB; min diag length, 24) in MEGA ver. 7.0. Bootstrap support values (≥50%) and gene names are shown.


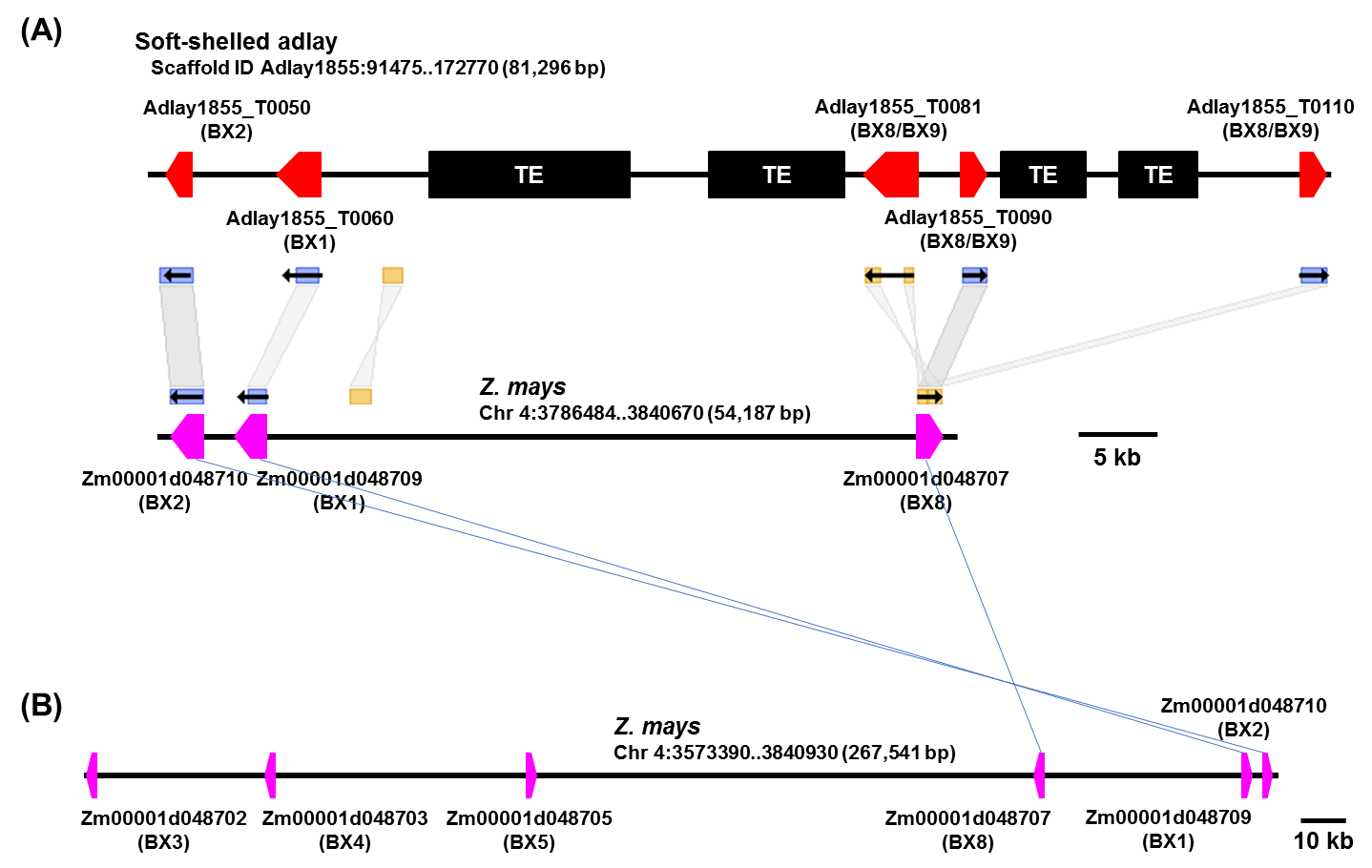


**Supplementary Figure S15**. Sequence level comparison of collinear genomic regions harboring BX genes in soft-shelled adlay (*C. lacryma-jobi* var. *ma-yuen* ‘Johyun’ in this study) and maize (*Z. mays*). Syntenic genomic sequences were searched by BLASTN and TBLASTN analyses (*E*-value cutoff of 1e-5) against maize genome sequence with BX genes of soft-shelled adlay as queries. Genomic organization such as gene position and order among genomic regions was determined based on the annotation information of each genome sequence. **(A)** Microsynteny between the collinear genomic sequences harboring BX genes in soft-shelled adlay and maize. Microsynteny was analyzed at the nucleotide level by local BLASTZ searches with default parameters (threshold MSPs of 10,000, gapped alignment of 10,000) and visualized by a BLAST viewer. Red and pink boxes indicate BX genes in soft-shelled adlay and maize, respectively. The gray bars connecting boxes between sequences indicate conserved sequences. **(B)** Genomic organization of maize 267.5 kb-genomic region harboring six BX genes. This information was retrieved from Ensembl plant genome database (<https://plants.ensembl.org/Zea_mays/Location/View?r=4:3573390-3840932;db=core>).


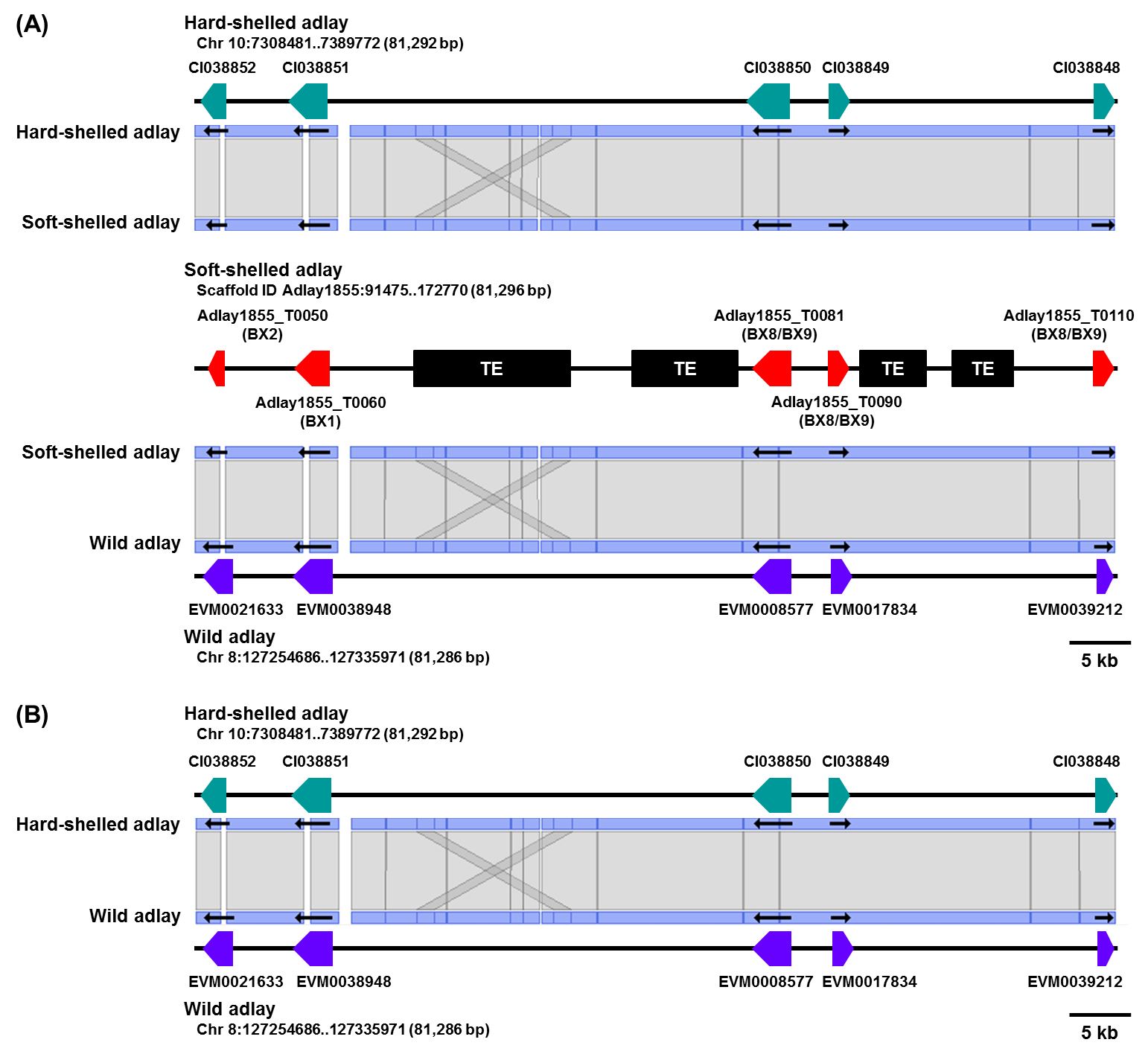


**Supplementary Figure S16**. Sequence level comparison of collinear genomic regions harboring BX genes in soft-shelled adlay (*C. lacryma-jobi* var. *ma-yuen* ‘Johyun’ in this study), hard-shelled adlay (*C. lacryma-jobi* var. *lacryma-jobi*, Liu et al., 2019), and wild adlay (*C. aquatica*, Guo et al., 2019). Collinear genomic sequences were searched by BLASTN analyses (*E*-value cutoff of 1e-10, megablast option) against two *Coix* genome sequences with genomic query sequence of soft-shelled adlay. Genomic organization such as gene position and order among collinear genomic regions was determined based on the annotation information of each genome sequence. **(A)** Microsynteny among the collinear genomic sequences harboring BX genes in soft-shelled adlay, hard-shelled adlay and wild adlay. **(B)** Microsynteny between the collinear genomic sequences harboring BX genes in hard-shelled adlay and wild adlay. Microsynteny was analyzed at the nucleotide level by local BLASTN searches with modified parameters (megablast option, *E*-value cutoff of 1e-06, minimum match length of 1,000 bp) and visualized by a BLAST viewer. Red boxes indicate BX genes in soft-shelled adlay and boxes of other colors indicate synteny-based orthologs identified in hard-shelled adlay and wild adlay. The gray bars connecting boxes between sequences indicate conserved sequences.

**Supplementary Figure S17**. Expression patterns of maize BX biosynthesis genes. Expression values (FPKM) of genes were retrieved using MaizeGDB qTeller tools (<https://qteller.maizegdb.org/>). Expression values (FPKM) were scaled per row (i.e., per gene) to visualize gene expression peaks among the different tissues, and the heatmap was generated using the R-package pheatmap ver. 1.0.12 (https://CRAN.R-project.org/package=pheatmap). Tissue names and gene symbols are shown at the bottom and on the right of the heatmap, respectively. a, mature leaf; b, internode 6 and 7; c, internode 7 and 8; d, primary root 5 days; e, secondary root 7-8 days; f, silks; g, mature female spikelets; h, mature pollen; i, endosperm 12 days after pollination (DAP); j, embryo 20 DAP; k, embryo 38 DAP.
